# CD6 is a target for cancer immunotherapy

**DOI:** 10.1172/jci.insight.145662

**Published:** 2021-03-08

**Authors:** Jeffrey H. Ruth, Mikel Gurrea-Rubio, Kalana S. Athukorala, Stephanie M. Rasmussen, Daniel P. Weber, Peggy M. Randon, Rosemary J. Gedert, Matthew E. Lind, M. Asif Amin, Phillip L. Campbell, Pei-Suen Tsou, Yang Mao-Draayer, Qi Wu, Thomas M. Lanigan, Venkateshwar G. Keshamouni, Nora G. Singer, Feng Lin, David A. Fox

**Affiliations:** 1 Division of Rheumatology,; 2 Department of Neurology, and; 3 Division of Pulmonary & Critical Care Medicine, University of Michigan, Ann Arbor, Michigan, USA.; 4 Case Western Reserve University,; 5 Division of Rheumatology, MetroHealth Medical Center, Cleveland, Ohio, USA.; 6 Department of Immunity and Inflammation, Lerner Research Institute, Cleveland Clinic, Cleveland, Ohio, USA.

**Keywords:** Immunology, Cancer immunotherapy, NK cells, T cells

## Abstract

Limitations of checkpoint inhibitor cancer immunotherapy include induction of autoimmune syndromes and resistance of many cancers. Since CD318, a novel CD6 ligand, is associated with the aggressiveness and metastatic potential of human cancers, we tested the effect of an anti-CD6 monoclonal antibody, UMCD6, on killing of cancer cells by human lymphocytes. UMCD6 augmented killing of breast, lung, and prostate cancer cells through direct effects on both CD8^+^ T cells and NK cells, increasing cancer cell death and lowering cancer cell survival in vitro more robustly than monoclonal antibody checkpoint inhibitors that interrupt the programmed cell death 1 (PD-1)/PD-1 ligand 1 (PD-L1) axis. UMCD6 also augmented in vivo killing by human peripheral blood lymphocytes of a human breast cancer line xenotransplanted into immunodeficient mice. Mechanistically, UMCD6 upregulated the expression of the activating receptor NKG2D and downregulated expression of the inhibitory receptor NKG2A on both NK cells and CD8^+^ T cells, with concurrent increases in perforin and granzyme B production. The combined capability of an anti-CD6 monoclonal antibody to control autoimmunity through effects on CD4^+^ lymphocyte differentiation while enhancing killing of cancer cells through distinct effects on CD8^+^ and NK cells opens a potential new approach to cancer immunotherapy that would suppress rather than instigate autoimmunity.

## Graphical abstract

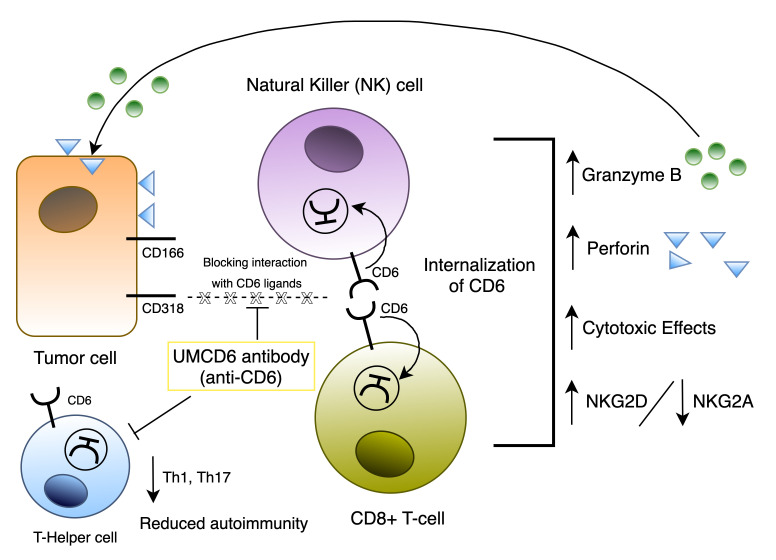

## Introduction

Checkpoint inhibitor therapy, directed at programmed cell death 1 (PD-1), PD-1 ligand 1 (PD-L1), or cytotoxic T lymphocyte–associated protein 4 (CTLA-4), has revolutionized cancer immunotherapy. However, cancers respond with varying efficacy to checkpoint inhibition, and many patients also experience severe autoimmune-related adverse events with these therapies (1). Therefore, additional targets are needed on cancer cells and lymphocytes that enhance immune cell elimination of tumors without engendering autoimmune toxicities through induction of lymphocyte self-reactivity. CD318 (CDCP1, TRASK, SIMA135, or gp140) is a cell surface glycoprotein that is widely expressed by cancer cells, and its degree of expression correlates with cancer aggressiveness and metastatic potential (2–4). Prior studies of CD318 have been limited to its intrinsic roles in cancer cell biology, but its possible participation in immune regulation has not to our knowledge been examined. Notably, we recently discovered that CD318 is a second ligand for the CD6 T cell surface glycoprotein (5).

CD6 is a 105 kDa to 130 kDa type I transmembrane glycoprotein belonging to the highly conserved scavenger receptor cysteine-rich superfamily (SRCR-SF) (6), almost exclusively expressed by lymphocytes, including most mature T cells and about 50% of NK cells (7). CD6 is also a receptor for CD166/activated leukocyte cell adhesion molecule (ALCAM) (8, 9). The interaction between CD6 and CD166 helps to stabilize the adhesive contacts established between T cells and antigen-presenting cells (APCs), as well as to optimize subsequent proliferative and differentiation responses (10–12).

We recently showed CD6 to be essential in murine models of multiple sclerosis (MS) (13), uveitis (14), and rheumatoid arthritis (RA) (15). In both *CD6^–/–^* mice and CD6-humanized mice treated with the mouse anti–human CD6 mAb UMCD6, striking reductions in clinical signs of disease, pathogenic Th1/Th17 responses, and inflammatory cell infiltration into the target organs were observed (13–15). Both known CD6 ligands, CD318 and CD166, participate in adhesion of T cells to fibroblast-like synoviocytes (FLS) derived from RA synovial tissue by engagement of distinct domains on CD6. Moreover, soluble CD318 (sCD318) is found in RA synovial fluid at levels higher than in normal or RA serum, and sCD318 is chemotactic for T cells at a concentration equal to this in vivo gradient (5).

In light of these recent observations, we have now tested the effects of interrupting the interactions between CD6 on lymphocytes with CD6 ligands on cancer cells on the ability of human lymphocytes to kill the cancer cells. Coculture experiments using a multiplexed time-lapse imaging system, including cell lines derived from human triple-negative breast cancer, non–small cell lung cancer, and prostate cancer, showed substantial enhancement of cancer cell death and reduced survival of cancer cells in the presence of UMCD6 and otherwise nonstimulated human lymphocytes. This effect was consistently more robust in vitro than the effect of either pembrolizumab or nivolumab, which are checkpoint inhibitor immunotherapies that are currently widely used in cancer treatment. We also demonstrate that augmentation of lymphocyte cytotoxicity by UMCD6 is due to direct effects of this mAb on NK cell and CD8^+^ cytotoxic T cells, including augmentation of the expression of the activating receptor NKG2D and decreased expression of the inhibitory NKG2A receptor. Moreover, UMCD6 exerted similar effects in vivo in a human breast cancer xenograft system in immunodeficient mice. Both in vitro and in vivo, UMCD6 is rapidly internalized and is therefore a nondepleting mAb.

These results indicate that CD6 is a promising new target for cancer immunotherapy. Because anti-CD6 has distinct effects on CD4^+^ cells that suppress autoimmunity, coupled with direct effects on CD8^+^ cells and NK cells that promote the killing of cancer cells, use of this approach to treat human cancer could avoid the troubling autoimmune complications frequently seen with currently available checkpoint inhibitors.

## Results

### High expression of CD318 on cancer cell lines.

**Figure 1:**
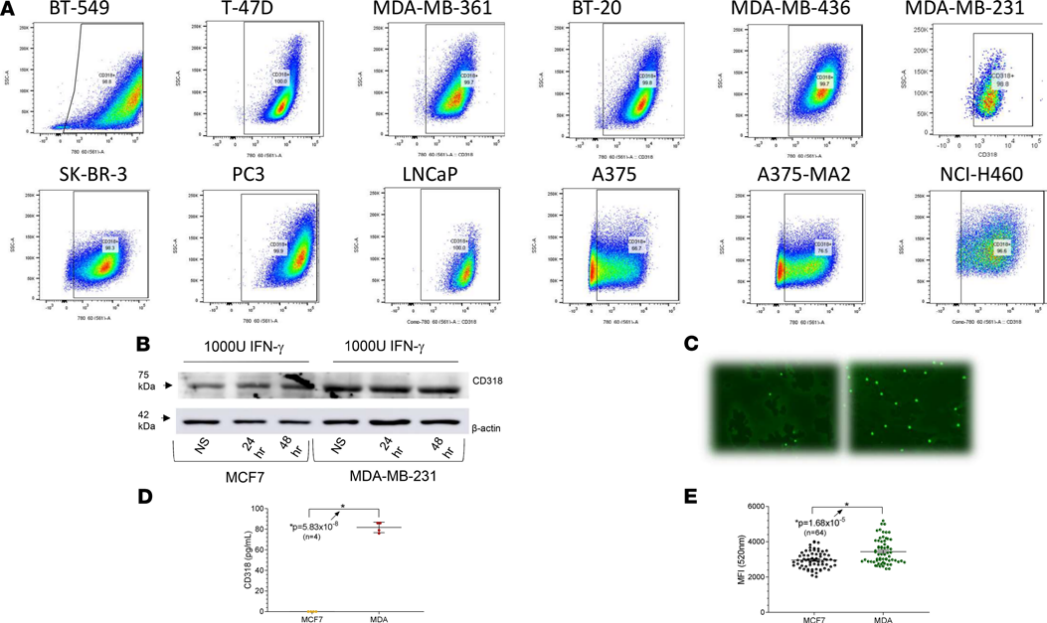
Expression of CD318 on multiple cancer cell lines. (**A**) Flow cytometry revealed robust expression of CD318 on the breast cancer lines BT-549, T-47D, MDA-MB-361, BT-20, MDA-MB-436, MDA-MB-231, and SK-BR-3; the prostate cancer lines PC3 and LNCaP; the melanoma cell lines A375 and A375-MA2; and the non–small cell lung cancer line NCI-H460. Tumor lines with low expression of CD318 included MCF7 (breast), UM-MEL-1 (melanoma), and HS587 (lung) — not pictured here. (**B**) FACS analysis was confirmed by immunoblotting of tumor cell lysates from MDA-MB-231 and MCF7 human breast cancer cells (HBCCs). IFN-γ had a negligible effect on CD318 expression on HBCCs, distinct from the previously observed induction of CD318 by IFN-γ on cultured synovial fibroblasts. (**C** and **D**) MDA-MB-231 and MCF7 HBCCs were plated in a 96-well plate at 20,000 cells per well, followed by addition of 50,000 CFSE-labeled T cells (*n* = 64). Adhesion was measured after 1 hour at 37°C as CFSE green fluorescence using a BioTek Synergy Plate Reader at 40× magnification. More lymphocytes bound to the CD318^+^ MDA-MB-231 cells than to the CD318^–^ MCF7 cells. (**E**) Soluble CD318 is shed from CD318^+^ cancer cells. ELISA for sCD318 in culture supernatants of CD318^–^ MCF7 cells and CD318^+^ MDA cells shows that HBCCs that express CD318 can also shed sCD318 into cell culture supernatants at concentrations previously shown to induce lymphocyte chemotaxis.

Multiple human cancer cell lines were analyzed by flow cytometry for expression of CD318, which was recently described as a second ligand of CD6 (Figure 1). The majority of malignant cell lines derived from patients with breast cancer, non–small cell lung cancer, prostate cancer, and melanoma were CD318^+^, several at high mean fluorescence intensity. The breast cancer cell line MCF7 (Supplemental Figure 1; supplemental material available online with this article; https://doi.org/10.1172/jci.insight.145662DS1) and the melanoma cell line UM-MEL1 (data not shown) had little or no surface CD318. All lines tested expressed moderate to high levels of CD166/ALCAM, a ligand of CD6 that is found on activated leukocytes, cancer cells, and many normal tissue cell populations (16) (Figure 1A). We confirmed the flow cytometry results by Western blot analysis of MDA-MB-231 (surface CD318^+^) and MCF7 (surface CD318^–^) breast cancer cells, and also tested the effect of IFN-γ, which induces expression of CD318 on non-neoplastic cells such as synovial fibroblasts (Figure 1B). Abundant CD318 was present in lysates of MDA-MB-231 compared with a smaller amount in MCF7 lysates, and IFN-γ did not alter expression of CD318 by these cells (Figure 1B and Supplemental Figure 1) or by other cancer cell lines (data not shown). Soluble CD318 was shed into the culture medium from the surface of CD318^+^ breast cancer cells (Figure 1E), as previously observed in cultures of RA synovial fibroblasts, and at concentrations shown to induce T cell chemotaxis (5). Moreover, T lymphocytes adhered in greater numbers to a CD318^+^ than a surface CD318^–^ breast cancer line (Figure 1, C and D).

### In vitro killing of breast and prostate cancer cells is enhanced by mAbs to CD6 or CD318.

To explore the possibility that CD6-CD318 could be a potential target for lymphocyte checkpoint inhibition, we used an IncuCyte imaging device to image cocultures of cancer cell lines and PBMCs or purified lymphocyte subsets, with or without anti-CD6 (UMCD6) or anti-CD318 (3a11) mAbs. As controls, we used mAb to lymphocyte function–associated antigen 1 (LFA-1)/CD11a/CD18, an isotype-matched anti-vWF mAb that does not bind to lymphocytes or cancer cells, and/or mouse IgG. Additionally, a nontoxic caspase reagent that fluorescently labels dying cells was added to proliferating cancer cells without the addition of immune cells or antibody, to monitor cancer cell growth and survival in culture.

**Figure 2:**
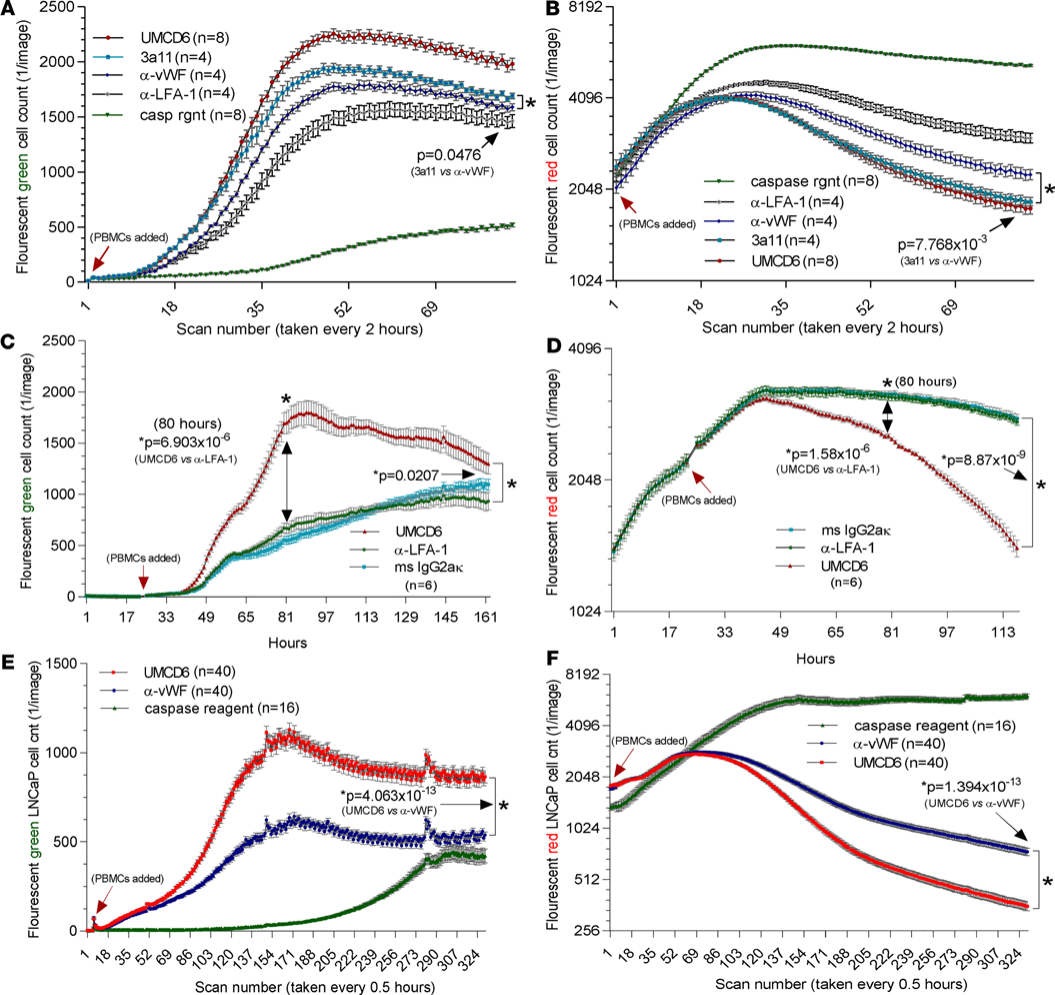
UMCD6 antibody enhances cancer cell killing by PBMCs. Tumor cells were cocultured and imaged using an IncuCyte system that recorded tumor cell number (**B**, **D**, and **F**, red fluorescence with *y* axis log_2_) and cell death (**A**, **C**, and **E**, green fluorescence, caspase sensitive with *y* axis linear). (**A** and **B**) CD318-expressing MDA-MB-231 cancer cells were plated in a 96-well plate with a seeding density of 20,000 cells with 50,000 PBMCs. Enhanced killing of MDA-MB-231 HBCCs by PBMC was observed in the presence of anti-CD6 (UMCD6) or anti-CD318 (3a11) for caspase expression compared with control antibodies (UMCD6 vs. anti-vWF *P* = 0.000623; UMCD6 vs. 3a11 *P* = 0.00401; 3a11 vs. anti-vWF *P* = 0.0476) and tumor cell survival (UMCD6 vs. anti-vWF *P* = 0.00223; UMCD6 vs 3a11 *P* = 0.4507; 3a11 vs anti-vWF *P* = 0.0078). (**C** and **D**) MDA-MB-231 cells were plated at a seeding density of 20,000 cells per well. 50,000 PBMCs were added at 22 hours. Before addition to the cocultures, PBMCs were incubated for 1 hour at 37°C with UMCD6 or mouse IgG control antibodies. (**E** and **F**) LNCaP prostate cancer cells were plated at a seeding density of 20,000 cells per well. 50,000 PBMCs were added to the LNCaP cell cultures at 4 hours. Before addition to the cocultures, PBMCs were incubated with UMCD6 or IgG control antibodies. MDA HBCCs and LNCaP cells displayed profound enhancement of clumping and caspase expression in cocultures in which PBMCs were exposed to UMCD6 (**A** and **C**). MDA and LNCaP cells also displayed inhibited growth in the wells containing UMCD6-treated PBMCs (**B** and **D**). Statistical significance (*P < 0.05) was initially achieved for MDA-MB-231 between the UMCD6- and anti–LFA-1 IgG–treated cocultures at 39 hours (green, caspase cell death) and 51 hours (red, survival). Similarly, differences in LNCaP cell death became significant at 8.5 hours (**E**) and at 43.5 hours for survival (**F**) between UMCD6 and anti-vWF IgG cocultures. See Supplemental Videos 1–3 for MDA-231 breast cancer cell killing.

We investigated whether blocking CD6 or CD318 would affect immune cell–mediated killing of multiple cancer cell lines and overall growth/survival of the cultured cancer cells. Preincubation with triple-negative breast cancer MDA-MB-231 cells with mAb 3a11 (mouse anti–human CD318) enhanced killing and inhibited survival of cancer cells compared with the control antibodies (anti-vWF and anti–LFA-1), but UMCD6 (anti-CD6) was more effective than anti-CD318 in augmenting cancer cell killing by PBMCs (Figure 2, A and B). Interpretation of the effects of anti-CD318 could be confounded by the potential for nonspecific triggering of antibody-dependent cellular cytotoxicity through opsonization of cancer cells by anti-CD318, in addition to interruption of the ability of CD318 to engage CD6. Moreover, CD318 might not be a suitable molecular target for in vivo immunotherapy, due to its expression on many types of stromal and epithelial cells. Therefore, subsequent experiments focused primarily on UMCD6 and not anti-CD318.

A similar robust induction of breast cancer cell death and overall decrease in the number of surviving cancer cells was observed with UMCD6 using PBMCs from a second donor (Figure 2, C and D) and in PBMCs cocultured with a prostate cancer line (Figure 2, E and F). Cancer cell cultures with caspase reagent only (no antibodies or PBMCs added) showed insignificant cancer cell death and rapid cancer cell proliferation (Figure 2 and Supplemental Figure 2).

### UMCD6 effectively mediates PBMC killing of non–small cell lung carcinoma and breast cancer cells compared with pembrolizumab and nivolumab in vitro.

**Figure 3:**
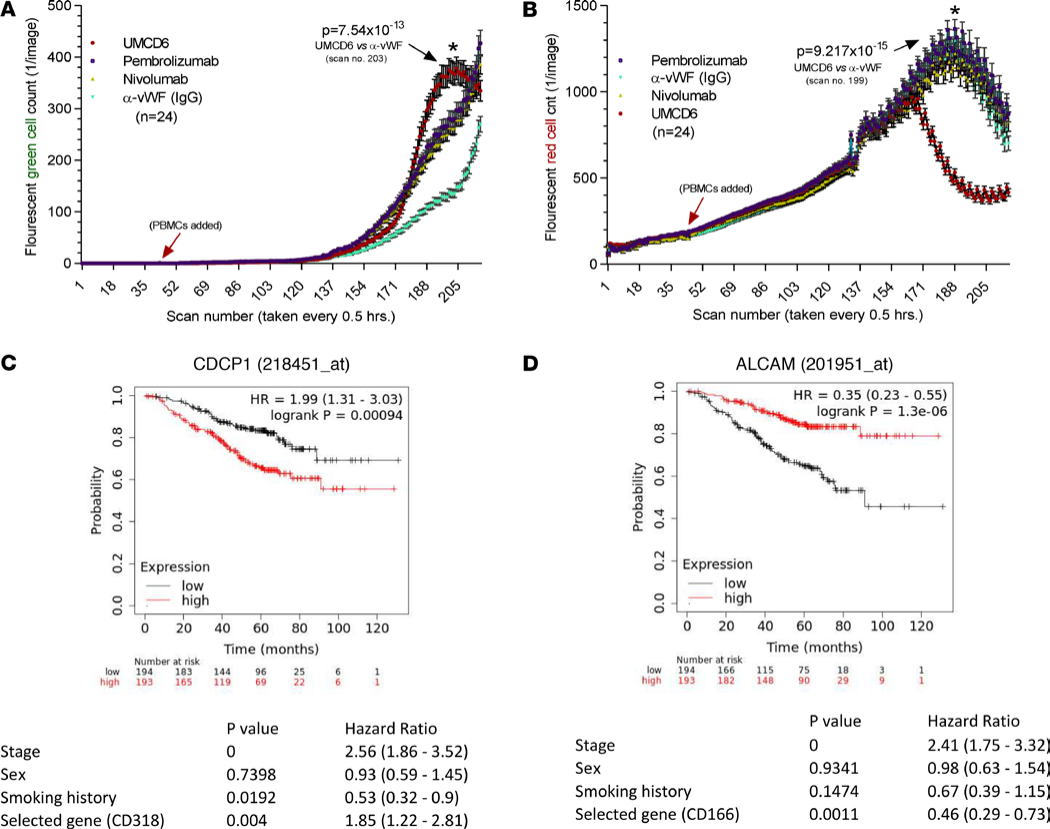
UMCD6 antibody enhances non–small cell lung cancer (NSCLC) cell killing by PBMCs. (**A**) Nuclear localized mKate2-transduced NCI-H460 cells were plated in a 96-well plate at 2000 cells per well. PBMCs (35,000) were added (*n* = 24 wells for each condition) at 22 hours (indicated by arrow). Before addition to the cocultures, PBMCs were incubated for an hour with UMCD6 (mouse anti–human CD6); mouse anti–human vWF; an IgG control antibody that did not bind to either PBMCS or the tumor cells; pembrolizumab; or nivolumab, all at 10 μg/mL. Tumor cell killing was measured in an IncuCyte cell imaging device as the number of NCI-H460 cells in each well expressing nuclear caspase (green fluorescence). NCI-H460 cells in cultures with UMCD6 showed profound clumping and caspase expression after 101.5 hours (scan no. 203) compared with the IgG control– or anti–PD-1–treated cocultures (data expressed as mean ± SEM; green fluorescence, caspase sensitive, with *y* axis linear). (**B**) Tumor cell survival was measured as the number of red fluorescing tumor cells remaining in culture (right panels, red fluorescence tumor cell survival, with *y* axis linear). The number of surviving tumor cells was significantly higher in the IgG, pembrolizumab, and nivolumab groups compared with the wells containing PBMCs that had been preincubated with UMCD6 (red line). (**C** and **D**) Multivariate survival analysis in patients with lung adenocarcinoma stratified according to cancer cell expression of the CD6 ligands CD318 and CD166/ALCAM. Expression of CD318 was assessed in a cohort of primary human lung adenocarcinoma patients (*n* = 387) (**C**). Overall survival analysis revealed that increased expression of CD318 strongly correlated with poor patient survival. However, CD166/ALCAM expression correlated with a better prognosis/longer patient survival (**D**).

We next evaluated the effectiveness of UMCD6 in enhancing killing of cancer cells representative of a cancer type, non–small cell lung carcinoma (NSCLC), in which PD-1/PD-L1–targeted checkpoint inhibitor immunotherapy is currently widely used. In these experiments we compared the effects of UMCD6 with those of pembrolizumab and nivolumab, agents that target the PD-1/PD-L1 checkpoint pathway. Freshly isolated human PBMCs were preincubated with UMCD6, pembrolizumab, nivolumab, or an anti-vWF control antibody. After an hour of incubation, treated PBMCs were added to NCI-H460 tumor cells with a caspase detection reagent, and monitored for cellular caspase expression (indication of immune cell killing) and tumor cell growth and survival, indicated by the number of red-fluorescing tumor cells remaining in coculture over time (Figure 3, A and B). As observed with the breast and prostate cancer cell lines, a substantial increase in NCI-H460 cell death occurred in the UMCD6-treated compared to the anti-vWF–treated cocultures. Moreover, the effects of UMCD6 on cancer cell death and survival were significantly stronger than the effects of either pembrolizumab and nivolumab, when used at identical concentrations (Figure 3 and Supplemental Figure 3). Thus, PBMCs pretreated with UMCD6 enhanced caspase expression and inhibited NSCLC cell survival to a much greater extent than checkpoint inhibitor immunotherapeutics currently used in clinical care. However, experimental conditions were not designed to optimize expression of PD-1.

Results from a univariate survival analysis in patients with lung adenocarcinoma stratified according to cancer cell expression of the CD6 ligands CD318 and CD166/ALCAM, using 2 different Affymetrix probes, showed that increased expression of CD318 correlated strongly with poor survival in patients with lung adenocarcinoma (*n* = 720) (Figure 3, C and D), consistent with previous observations (17). Conversely, lung adenocarcinoma expression of CD166/ALCAM correlated with an improved prognosis (Figure 3, C and D).These results suggest that CD6 interactions with CD6 ligands that are expressed on cancer cells have an important influence on clinical outcomes of cancer patients and point to the CD6/CD6 ligand axis as a potential new therapeutic target in cancer treatment.

**Figure 4:**
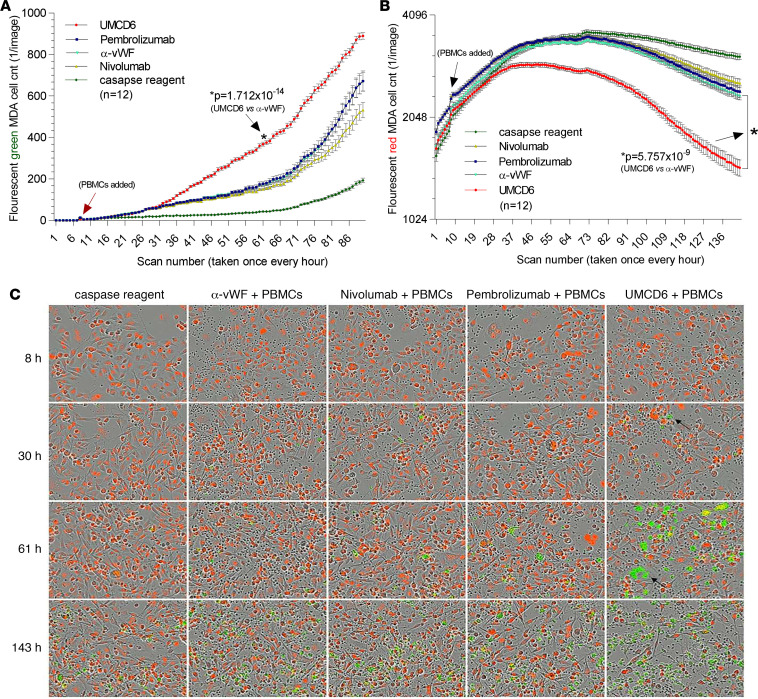
Effect of UMCD6 on breast cancer cell killing by PBMCs compared with effects of checkpoint inhibitor immune therapeutics directed at the PD-1/PD-L1 axis. (**A** and **B**) MDA-MB-231 tumor cells were plated in a 96-well plate at 20,000 cells/well. 50,000 PBMCs were added to the MDA-MB-231 cell cultures at 8 hours. Before addition to the cocultures, PBMCs were incubated for 1 hour at 37°C with UMCD6, vWF-IgG, or the same concentration of pembrolizumab or nivolumab, all at 10 μg/mL. Cancer cell killing was measured by the number of breast cancer cells present in each well (red fluorescence, tumor cell survival, with *y* axis log_2_) and cell death (green fluorescence, caspase sensitive, with *y* axis linear). Peak killing of cancer cells occurred around 61 hours, at which time MDA-MB-231 cells displayed profound caspase expression compared with the anti-vWF–treated wells (**A**; *P* = 1.712 × 10^–14^ at 61 hours). Statistical significance for caspase expression was initially achieved for MDA-MB-231 cell death at 31 hours for UMCD6 versus pembrolizumab and nivolumab — and at 30 hours for UMCD6 versus control IgG. MDA-MD-231 cells also showed inhibited growth and survival when exposed to the UMCD6-treated PBMC compared with the IgG control group from the beginning of the experiment that persisted and increased through 143 hours (**B**; UMCD6 vs. anti-vWF *P* = 5.757 × 10^–9^, UMCD6 vs. pembrolizumab *P* = 7.260 × 10^–8^, UMCD6 vs. nivolumab *P* = 7.186 × 10^–10^ at the final time point). (**C**) Single images of tumor cells in coculture with PBMCs and various antibody treatments. Hour 30 shows a coculture of PBMCs (small, round black cells) with dying tumor cells expressing caspase in the plate wells treated with UMCD6 (arrow). By 61 hours, wells treated with UMCD6 showed significantly more tumor cells with pronounced caspase expression that contained fewer tumor cells compared with cocultures treated with pembrolizumab, nivolumab, anti-vWF, or caspase reagent control culture. All images were taken at 20× magnification.

The superiority of UMCD6 in stimulating cancer cell killing by PBMCs was not confined to lung cancer but was also readily demonstrated using a breast cancer line (Figure 4). Photomicrographic images taken at 4 time points during a real-time kinetic immune cell cancer killing assay from the IncuCyte system show caspase expression by human breast cancer cells (fluorescent green) in coculture with PBMCs preincubated with UMCD6 as early as 30 hours — which increased significantly at 61 hours and beyond (Figure 4, A and C). There was a notable attrition of fluorescing red tumor cells in this coculture at 61 hours through the end of the assay, an effect that was less evident in cocultures treated with pembrolizumab, nivolumab, or control antibodies (Figure 1B).

### UMCD6 directly activates cytotoxic lymphocytes.

**Figure 5:**
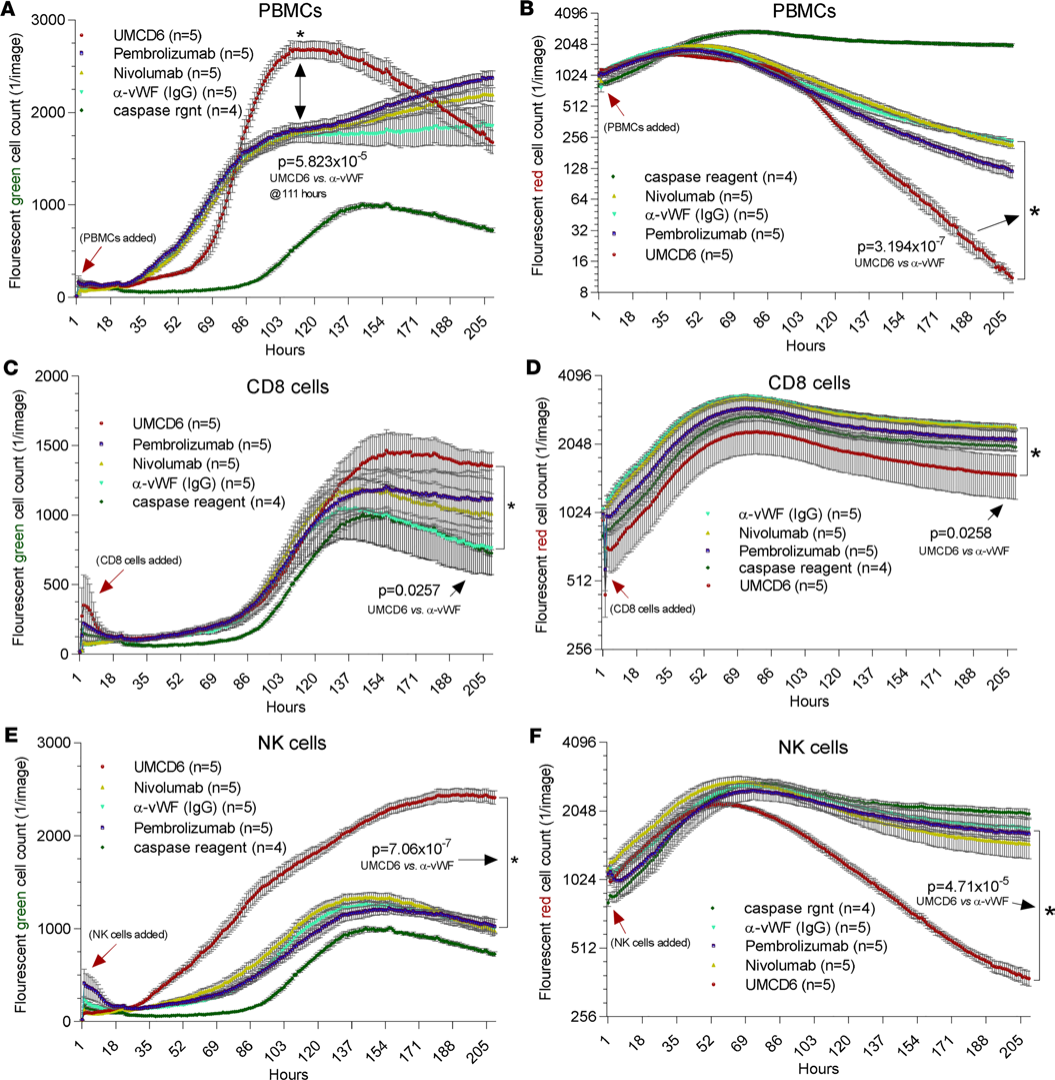
CD6^+^ NK cells treated with UMCD6 are highly effective at killing MDA-MB-231 tumor cells. (**A** and **B**) Tumor cell killing assays were set up using 50,000 immune cells and 20,000 MDA-MB-231 HBCCs and antibodies at 10 μg/mL. PBMCs preincubated with UMCD6 killed tumor cells much more effectively than PBMCs preincubated with IgG control antibody, pembrolizumab, or nivolumab (**A**; red fluorescence, tumor cell survival, with *y* axis log_2_) and cell death (**B**; green fluorescence, caspase sensitive, with *y* axis linear). (**C** and **D**) Isolated CD8^+^ cells showed enhanced killing and lower tumor cell survival in cocultures with UMCD6 compared with the other antibodies. (**E** and **F**) Only UMCD6-induced tumor cell killing by purified NK cells.

Since CD8^+^ T cells and a subset of human NK cells express CD6 (7, 8), we next asked whether either or both of these populations could be activated by UMCD6 to manifest augmented killing of cancer cells. Indeed, UMCD6 enhanced cancer cell killing by purified human CD8^+^ or NK cells (Figure 5). As expected, PBMCs treated with UMCD6 showed enhanced killing and reduced survival of breast cancer cells in coculture. Pembrolizumab and nivolumab also showed enhanced PBMC-mediated killing activity and reduced viability of cancer cells, but to a lesser degree than observed with UMCD6 (Figure 5, A and B). Similarly, UMCD6-treated CD8^+^ lymphocytes, isolated from PBMCs by negative selection, showed greater cancer cell killing with UMCD6 compared with pembrolizumab or nivolumab (Figure 5, C and D). Notably, purified CD56^+^ NK cells showed distinct and robust effects on cancer cell killing and survival curves only in the presence of UMCD6 (Figure 5, E and F). Neither pembrolizumab nor nivolumab had any effect on NK cell killing of cancer cells, possibly due to the low expression of PD-1 on nonactivated NK cells. These results point to unique mechanisms of action of UMCD6 compared with other checkpoint inhibitors in activating lymphocyte subsets, especially NK cells.

Because activation of T cells by mAbs such as anti-CD3 can be accompanied by internalization of their target surface structures (18), we asked whether UMCD6 induced internalization of CD6. Indeed, UCMD6 quickly led to capping of CD6, with subsequent clearing of CD6 from the cell membrane over 6 hours (Supplemental Figure 4). UMCD6-treated lymphocytes remained CD6^–^ for several days, consistent with previous observations in vivo in CD6-humanized mice, in which a large population of CD6^–^ lymphocytes appeared after administration of UMCD6 (15).

### UMCD6 enhances PBMC killing of human breast cancer cells in vivo.

**Figure 6:**
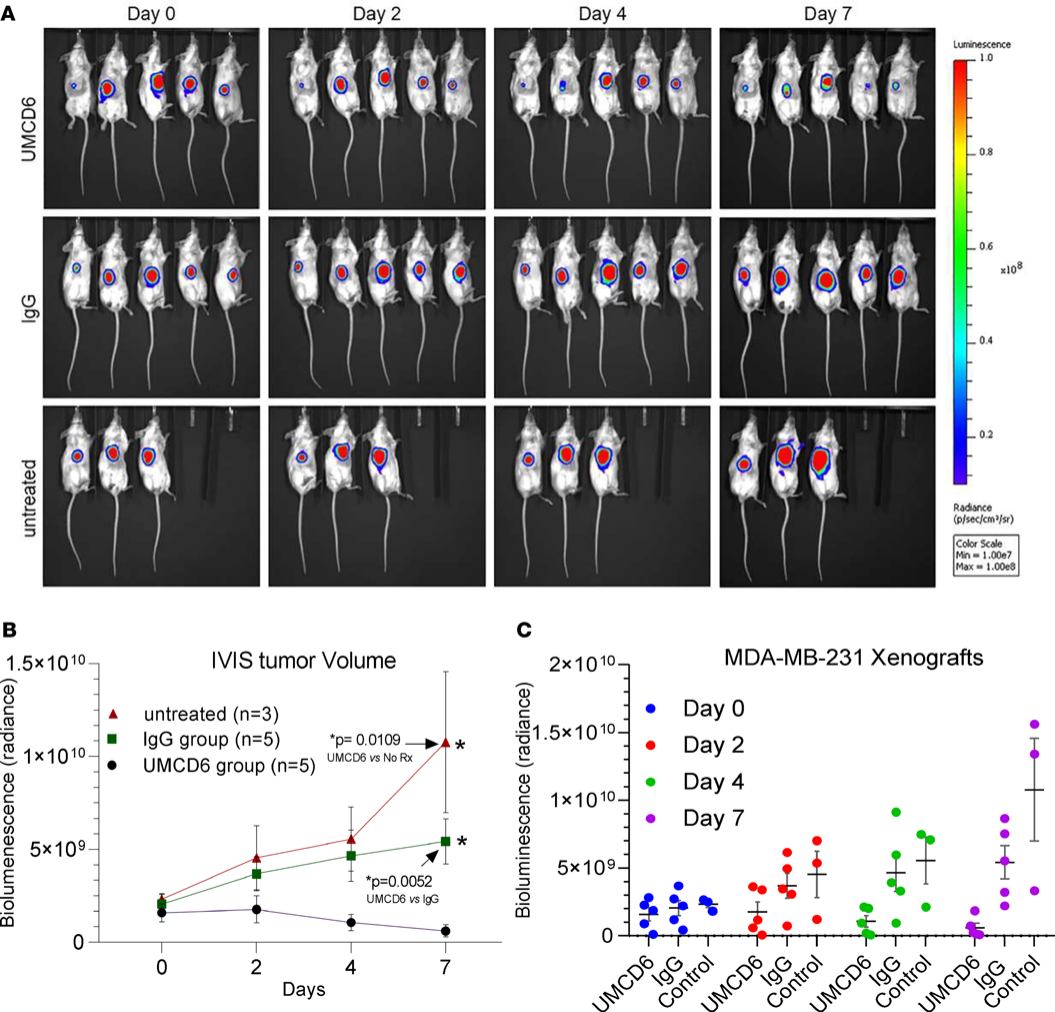
UMCD6 reduces tumor size in SCID beige mice. (**A**) HBCCs (MDA 5 × 10^6^ cells) were inoculated s.c. in the ventral aspect of the abdomen of female SCID beige mice. Once tumors reached about 100 mm^3^, some mice were administered 12 × 10^6^ human PBMCs by tail vein (considered day 0). The next day, mice that had received PBMCs were injected i.p. with 0.4 mg control IgG or UMCD6. Mice not administered PBMCs received no antibodies (untreated). Tumors were measured by IVIS (in vivo imaging) thereafter. (**B** and **C**) The effect of UMCD6 on tumor volume can be seen on days 4 and 7 after UMCD6 administration (*P < 0.05) compared with both the IgG and no-treatment groups. Data represent mean of 3–5 animals ± SEM. 2-tailed *t* test on day 7: UMCD6 vs. IgG, *P* = 0.0052; UMCD6 vs. untreated, *P* = 0.0109. 2-tailed *t* test on day 4: UMCD6 vs. IgG, *P* = 0.0038; UMCD6 vs. untreated, *P* = 0.0177.

To evaluate the therapeutic efficacy of UMCD6 in vivo, we next generated a xenograft mouse model of triple-negative breast cancer by subcutaneously injecting 5 × 10^6^ luciferase-labeled MDA-MB-231 cells into the right flank region of immunodeficient SCID beige mice (Figure 6A). Tumor proliferation was monitored by bioluminescence imaging. Twenty-six days after tumor implantation, when tumors reached volumes of at least 100 mm^3^, 10 mice received an intravenous injection of 1.2 × 10^7^ PBMCs via the tail vein, while the 3 other mice received PBS. The following day, mice that had received PBMCs were i.p. injected with a single dose of UMCD6 or IgG control (400 μg/mouse). As measured by bioluminescence imaging, tumor growth was significantly reduced in mice that received UMCD6 compared with control IgG on day 4 (*P* = 0.038) and day 7 (*P* = 0.0052) after antibody injection (Figure 6, B and C).

Because sCD318 is chemotactic for T cells (5), we investigated whether cytotoxic lymphocytes migrated to tumor sites. At 10 days after the injection of PBMCs, tumors were harvested and frozen sections were immunostained for tumor-infiltrating lymphocytes. Both CD3^+^ T cells and CD56^+^ NK cells were found exclusively in mice that had received an intravenous injection of PBMCs (Supplemental Figure 5). Although NK cells do not always infiltrate tumors, their presence in tumor biopsies has been positively associated with increased survival and better prognosis in several cancer types (19–23). Both T cells and NK cells appeared activated, and were more abundant in tumors from mice treated with UMCD6 than in IgG control mice. We also determined the tumor cell density in each tumor based on the number of mKate2-expressing cells. Treatment with UMCD6 reduced the number of remaining tumor cells per field compared with controls (Supplemental Figures 5 and 6). These in vivo results indicating the participation of both NK and T cells in the antitumor effects of UMCD6-stimulated lymphocytes are consistent with those seen in vitro.

### UMCD6 induces upregulation of NKG2D on NK and CD8^+^T cells.

**Figure 7:**
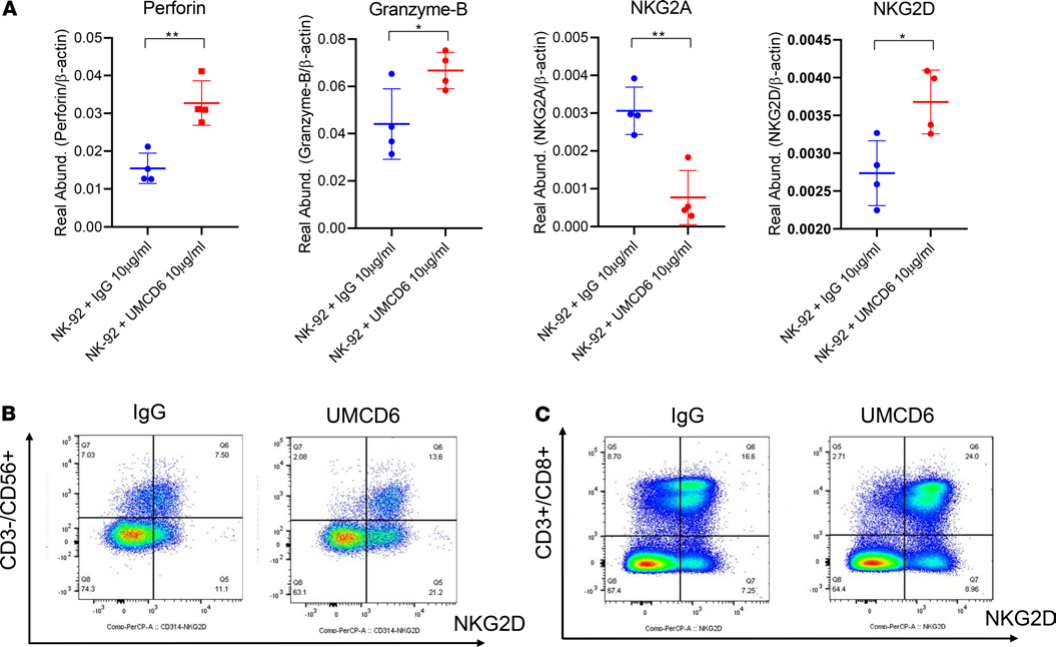
UMCD6 induces upregulation of NKG2D on NK and CD8^+^ T cells. (**A**) NK-92 cells were incubated with 10 μg/mL of IgG or UMCD6 and harvested after 4 hours. Real-time PCR revealed significantly higher levels of mRNA for the activating receptor NKG2D, as well as perforin and granzyme B upon incubation with UMCD6, while mRNA for the inhibitory receptor NKG2A was downregulated. Data are expressed as mean ± SD; *P < 0.05. (**B** and **C**) Representative flow cytometry plots showing NKG2D-positive NK and CD8^+^ T cells. PBMCs from 6 donors (*n* = 6) were isolated as described in Methods and subsequently cultured with 10 μg/mL IgG or UMCD6. Increase of cell surface expression of NKG2D was evident 72 hours after treatment with UMCD6 on NK cells (gated on CD3^–^CD56^+^ cells) (**B**) and CD8^+^ T cells (gated on CD3^+^CD8^+^ cells) (**C**).

NK cell and CD8^+^ T cell functionality is regulated by a balance between a variety of activating and inhibitory receptors, including CD94/NKG2A (inhibitory) and CD314/NKG2D (activating). Once activated, NK cells and CD8^+^ T cells exhibit cytotoxicity and cytokine production against tumor cells and virus-infected cells. To investigate the mechanisms by which CD6^+^ human NK cells are stimulated by UMCD6 to kill neoplastic cells, we asked whether UMCD6 affected the expression of various activating and inhibitory receptors on a human NK-cytotoxic cell line (NK-92). Interestingly, NKG2A mRNA levels were reduced in UMCD6-treated NK-92 cells 4 hours after incubation with UMCD6, while NKG2D expression was upregulated. A significant enhancement of perforin and granzyme B expression was also observed upon activation with UMCD6, confirming that CD6 plays an important mechanistic role in NK cell activation (Figure 7A).

In addition to NK-92 cells, PBMCs from 6 donors were also used to study the influence of UMCD6 on expression levels of these 4 key molecules by both NK and CD8^+^ T cells. The patterns of alteration of expression of these molecules by UMCD6 were similar to those seen with NK-92 cells (Figure 7B and Supplemental Figure 7). Upon activation with UMCD6, NK cells upregulated expression of NKG2D by 48 and 72 hours, whereas downregulation of NKG2A was observed (*P* < 0.05). Granzyme B expression was also upregulated by 48 hours (*P* < 0.05), and perforin expression was upregulated by 48 and 72 hours (*P* < 0.05). UMCD6 also altered the expression levels of NKG2A, NKG2D, granzyme B, and perforin by CD8^+^ T cells from 5 of 6 healthy blood donors, similar to the results seen with NK cells from all 6 donors.

## Discussion

The data in this report establish that the anti-CD6 mAb UMCD6 powerfully stimulates the ability of human lymphocytes to kill cancer cells of multiple types. We observed direct effects of UMCD6 on the killing ability of purified CD8^+^ T cells and purified NK cells. In contrast to currently employed checkpoint inhibitor cancer immunotherapies such as pembrolizumab and nivolumab, CD4^+^ T cells are not required for this effect of UMCD6. However, UMCD6 does have important effects on activation and differentiation of CD4^+^ cells that underlie the beneficial results of its use in animal models of human autoimmune diseases.

In both *CD6^–/–^* mice and in CD6-humanized mice treated with anti-CD6, robust protective effects were seen in mouse models of multiple sclerosis (13), autoimmune uveitis (14), and RA (15). The therapeutic benefit was manifested as amelioration of clinical indicators of disease, attenuation of the immune cell infiltrates into the target organs, and marked reduction of the Th1 and/or Th17 immune responses that are essential to these conditions. Initial stages of lymphocyte activation were not inhibited by genetic absence of CD6 or use of UMCD6 in the CD6-humanized mice, and these mice did not become lymphopenic when UMCD6 was administered (15). Lack of T cell depletion is attributable to the rapid internalization of CD6 upon binding of UMCD6 to the cell surface, and abundant CD3^+^CD6^–^ T cells were therefore detected in vivo after treatment with anti-CD6. Following internalization of CD6 by UMCD6, recovery of CD6 surface expression is delayed for at least several days. Therefore, the enhanced killing of cancer cells in our experiments occurred with CD8^+^ lymphocytes and NK cells that were CD6^–^ and unable to bind either CD6 ligand displayed on the cancer cell surface.

The known cell surface ligands of CD6 are CD166/ALCAM and CD318, also known as CDCP1 or TRASK (5). Either or both ligands are widely expressed by human cancers (24, 25), and we have not encountered a cancer cell line that lacks strong expression of at least one of these molecules. We previously observed that CD318 is shed from RA FLS and accumulates in a soluble form in RA synovial fluid at levels higher than found in normal or RA sera (5). At a concentration equal to this in vivo gradient, sCD318 is chemotactic for CD6^+^ lymphocytes. Like FLS, CD318^+^ cancer cells shed CD318 into their culture medium and are likely to also do this in vivo. For this reason, the in vivo breast cancer experiment was designed so that the infused human lymphocytes would have an opportunity to respond to sCD318 by migrating into the tumor microenvironment, and the injection of UMCD6 was therefore given 1 day later than the infusion of human PBMCs.

The heightened ability of UMCD6-treated CD6^–^ lymphocytes to kill cancer cells implies that CD6 ligands on the cancer cells deliver a negative signal to CD8^+^ and NK cytotoxic lymphocytes. Whether CD166 and CD318 are equally potent in this way is not known, but CD318 appears to be more broadly associated with a worse clinical outcome in human cancers (26–28). In non–small cell lung cancer for example, high expression of CD318 associates with poor outcome (29), while the opposite is true for CD166 (30). Experiments that manipulate the expression of these ligands on various cancer cell lines may be useful in addressing this issue. In autoimmune diseases, these ligands, which engage distinct domains of CD6, can have opposing effects. Thus, *CD318^–/–^* mice reproduced the phenotype of attenuated disease seen in *CD6^–/–^* mice in the mouse experimental autoimmune encephalomyelitis (EAE) model of MS (13), but mice lacking CD166 experienced exacerbation of EAE (31).

The hypothesis that cancer cell ligands of CD6 impair the function of cytotoxic lymphocytes is supported by experiments in which forced expression in vivo of a soluble form of CD6 was successfully employed as an anticancer strategy in mice (32). We observed enhancement of lymphocyte-mediated killing of cancer cells by a mAb against CD318, a CD6 ligand, which may represent a combination of nonspecific antibody-dependent cellular cytotoxicity and blockade of negative signals conveyed to lymphocytes from cancer cells arising from engagement of CD6 by CD318. Interruption of negative signals from cancer cells by UMCD6 does not, however, exclude the possibility that UMCD6 may also have a direct activating effect on T cells and NK cells.

NK cells are receiving increased attention recently as potential agents for cancer immunotherapy. Multiple structures on the NK cell surface can participate in activation of the NK cytotoxic program, while others have regulatory roles (33). To our knowledge, the importance of CD6 in NK cell activation has hitherto not been appreciated, perhaps due to the absence of CD6 on mouse NK cells, in contrast to its expression on about 50% or more of human NK cells (7). Our data demonstrate that UMCD6 can alter the balance of expression of activating and inhibitory receptors, and/or cytotoxic effector molecules on both NK and CD8^+^ lymphocytes. The changes observed thus far do not exclude potential roles of other alterations in surface structures and metabolic pathways that could be induced in cytotoxic lymphocyte populations by UMCD6.

Another anti-CD6 mAb has been used successfully in the treatment of psoriasis and thus far has an excellent safety record, pointing to the feasibility of testing anti-CD6 mAbs in the treatment of cancer as well as other autoimmune diseases (34, 35). In assessing UMCD6 as a new candidate cancer immunotherapy agent, its consistently superior stimulation of cancer killing by lymphocytes in vitro, compared with either pembrolizumab or nivolumab, is notable but will need confirmation in vivo, and use of cells from patients with cancer, which may express higher levels of PD-1 than cells from healthy subjects. The distinct mechanism of action of UMCD6 points to the potential for additive or synergistic effects of combination strategies directed at both CD6 and targets of currently used checkpoint inhibitors.

MHC class I chain–related molecules (MICA and MICB) are well-known ligands for the activating receptor NKG2D on NK and CD8^+^ T cells. Because MICA and MICB are highly expressed in a wide variety of tumor cells, targeting the CD6/CD318 axis with UMCD6 represents a broadly applicable approach to cancer immunotherapy that boosts cancer cell killing by multiple downstream effects on cytotoxic lymphocyte gene expression and effector function. The data regarding UMCD6-induced changes in gene expression of key cytotoxic cell effector molecules and activation or inhibition of cell surface receptors reveal CD6 as a molecule critical for controlling the activation state and function of human cytotoxic lymphocytes.

Our experimental systems used lymphocytes that are not autologous to the cancer cell lines with which they were cocultured. Nevertheless, our results are not explained by alloreactivity, for the following reasons. First, killing of cancer cells began far earlier in cocultures than would be consistent with development of an allogeneic response. Second, far less killing occurred in the absence of UMCD6, or in the presence of various control mAbs or IgG. Third, CD4^+^ lymphocytes, which are necessary for allosensitization, were not required and are likely irrelevant to the observed killing. Finally, NK cell function is not based on alloreactivity.

Perhaps of greatest importance, however, is the dual effect of UMCD6 in both suppressing autoimmune diseases through its effects on differentiation of effector CD4^+^ cell subsets and activating the anticancer cytotoxic properties of CD8^+^ and NK cells. This dual effect creates the potential for an approach to cancer immunotherapy that would, distinct from currently available checkpoint inhibitors, suppress rather than instigate serious autoimmune diseases, thus overcoming the major current limitation to the success of checkpoint inhibition in the treatment of human cancer.

## Methods

### Cell lines and cell culture.

The following human cancer cell lines were used in live cell imaging to assess immune cell killing of tumor cells: MDA-MB-231 (HTB-26), triple-negative (i.e., ER^−^PR^−^HER2^−^) epithelial breast carcinoma; NCI-H460 (HTB-177), large cell lung carcinoma; and LNCaP (HTB-1740), androgen-sensitive prostate adenocarcinoma. Cell lines were purchased from the ATCC and were cultured in reduced-riboflavin conditions using CMRL-1066 (Sigma-Aldrich) supplemented with 10% heat-inactivated FBS (HyClone) and 1% antibiotic-antimycotic solution (Gibco Life Technologies). Cells were maintained at 37°C in 5% CO_2_, and adherent cells were detached with trypsin (0.25%-EDTA, HyClone) for passaging and further culture. NK-92 cells were also purchased from ATCC and maintained in MEM-α containing 12.5% FBS, 12.5% horse serum (Gibco Life Technologies), 100 IU/mL IL-2 (R&D Systems), 0.2 mM inositol (Sigma-Aldrich), 0.02 mM folic acid (Sigma-Aldrich), and 0.1 mM mercaptoethanol (Gibco Life Technologies). Other cancer cell lines were screened for expression of CD318 (Table 1) and were grown in RPMI-1640 (HyClone) supplemented with 10% heat-inactivated FBS (HyClone) and 1% antibiotic-antimycotic solution (Gibco Life Technologies).

### Antibodies.

UMCD6, a mouse anti-human mAb that targets membrane distal domain 1 of CD6, was generated in our laboratory (36). UMCD6 was affinity purified and desalted by column chromatography using protein G and dextran per the manufacturer’s instructions (Thermo Fisher Scientific). The 3a11 mAb recognizes CD318 and was developed in our laboratory using IFN-γ–treated HBL100 cells (16). 3a11 was used for immune killing assays and Western blotting. Additionally, a second antibody against CD318 was purchased from BioLegend (clone CUB1) and was also used for flow cytometry analysis. Pembrolizumab and nivolumab (anti–PD-1 mAbs), used for immune killing assays, were obtained from Merck and Bristol Myers Squibb respectively. Mouse anti–human vWF (37) and mouse anti–human LFA-1 (CD11a/CD18) were obtained from the Hybridoma Core Facility at the University of Michigan.

The following human antibodies were purchased commercially and were used for immunofluorescence staining: Alexa Fluor 488 anti–human CD56 (BioLegend, clone 5.1H11), anti-CD3 (BioLegend, clone HIT3a), Alexa Fluor 488–conjugated goat anti-mouse IgG secondary antibody (Jackson ImmunoResearch Laboratories Inc., AB2338840), Alexa Fluor 488–conjugated donkey anti-mouse IgG secondary antibody (Thermo Fisher Scientific), and FITC-conjugated mouse IgG isotype control antibody (BioLegend, clone HP6017).

The following antibodies were used for flow cytometry analysis: For surface staining: APC anti–human CD56 (BioLegend, clone 5.1H11), FITC anti–human CD56 (BioLegend, clone HCD56), APC/Cyanine7 anti–human CD8a (BioLegend, clone RPA-T8), PE/Cyanine7 anti-human CD3 (BioLegend, clone HIT3a), PE-conjugated anti–human NKG2A (R&D Systems, FAB1059P), PerCP/Cyanine5.5 anti–human CD314 (NKG2D) (BioLegend, clone 1D11), FITC anti–human CD6 (BioLegend, clone BL-CD6), APC anti–human CD16 (BioLegend, clone 3G8); for intracellular staining: FITC anti–human/mouse granzyme B (BioLegend, clone GB11), PE anti–human/mouse granzyme B (BioLegend, clone QA16A02), PerCP/Cyanine5.5 anti–human perforin (BioLegend, clone B-D48), and APC anti–human perforin (BioLegend, clone B-D48).

### Flow cytometry.

MDA-MB-231, MDA-MB-361, MDA-MB-436, BT-20, BT-549, MCF7, T-47D, and SK-BR-3 breast cancer cells; A375, A375-MA2, and UM-MEL-1 melanoma cancer cells; PC3 and LNCaP prostate cancer cells; and NCI-H460 lung cancer cells were used to determine CD318 expression by flow cytometry. Briefly, cells were incubated with Fc receptor blocking solution (BioLegend) and stained with purified anti–human CD318 antibody (BioLegend, clone CUB1) in FACS buffer (PBS + 2% FBS + 2 mM EDTA) for 30 minutes on ice. Cells were subsequently washed in 1× PBS and stained using Alexa Fluor 488–conjugated donkey anti-mouse IgG secondary antibodies (Thermo Fisher Scientific) for 30 minutes on ice. Viability was assessed by staining with Zombie Violet (BioLegend), and cells were analyzed by flow cytometry at the University of Michigan Flow Cytometry Core on a BD Fortessa (Becton Dickinson). Analysis of flow cytometry data was performed using FlowJo software (Tree Star).

To determine the expression of NKG2A and NKG2D, we incubated PBMCs with Fc receptor blocking solution for 10 minutes at room temperature, followed by incubation with fluorescent antibodies against NKG2A (R&D Systems, FAB105P-025), NKG2D (BioLegend, 320817), CD56 (BioLegend, 318303), CD8 (BioLegend, 301016), CD3 (BioLegend, 300311), CD16 (BioLegend, 302011), and CD6 for 30 minutes on ice. For the intracellular staining of granzyme B and perforin, cells were first stained for anti-CD56, anti-CD3, and anti-CD8 and then fixed and permeabilized with FIX & PERM solution (Invitrogen, 00-833-56) for 45 minutes at room temperature. Cells were subsequently incubated with either anti–granzyme B or anti-perforin for 30 minutes at room temperature. Finally, cells were resuspended in 200 μL FluoroFix buffer (BioLegend) and analyzed by flow cytometry on a BD FACSCanto II at the University of Michigan.

### Western blotting.

Cell lysates were obtained from breast cancer cell lines MDA-MB-231 and MCF7 before and after stimulation with 1,000 U/mL human IFN-γ. Equal amounts of protein (15 μg per lane) were separated by Tris-Glycine SDS-PAGE and electroblotted onto nitrocellulose membranes. CD318 proteins were detected using anti-CD318 mAb 3a11 at 10 μg/mL, while β-actin (Sigma Aldrich) was used as a loading control. Bands were imaged on an Amersham Imager 600RGB (GE Healthcare), and quantification was performed using GelQuant.NET (BiochemLab Solutions).

### RNA extraction and quantitative real-time RT-PCR analysis.

1 × 10^6^ NK-92 cells were treated with 10 μg/mL of either UMCD6 or IgG and collected after 4 hours. Total RNA from treated NK-92 cells was extracted using Direct-zol RNA Miniprep (Zymo Research), and cDNA synthesis was carried out using A Verso cDNA Synthesis Kit (Thermo Fisher Scientific). The following primers were used for RT-PCR: NKG2A F 5′-ACTCATTGCTGGTACCCTGGG-3′, NKG2A R 5′-GAGGACAAGGCTGTGCTGAAG, NKG2D F 5′-TTCAACACGATGGCAAAAGC-3′ NKG2D R 5′-CTACAGCGATGAAGCAGCAGA-3′, perforin F 5′-GCTGGACGTGACTCCTAAGC-3′, perforin R 5′-GATGAAGTGGGTGCCGTAGT-3′, granzyme B F 5′-GCAGCCTTCCTGAGAAGATG-3′, granzyme B R 5′-CCGCACCTCTTCAGAGACTT-3′.

Quantification of mRNA expression was performed using the primers above and SYBR Green PCR Master Mix Reagent (Thermo Fisher Scientific) on a ViiA 7 Real-Time PCR System (Applied Biosystems). Triplicate measurements were performed for each sample. Real abundance for each gene was calculated using the ΔΔCT method, and β-actin was used as an internal standard for normalization.

### ELISA of sCD318.

Cell supernatants from MCF7 and MDA-MB-231 breast cancer cell lines were collected for the measurement of sCD318 by an ELISA kit (R&D Systems) following the manufacturer’s protocol.

### Lentivirus transduction.

To develop cell lines with nuclear fluorescence for live imaging, lentiviral stocks were developed by the University of Michigan Vector Core using an mKate2 2X nuclear localization fusion construct. Tumorigenic MDA-MB-231, NCI-H460, and LNCaP cells were seeded at 3 × 10^5^ cells/well on 6-well plates (Corning) overnight. The following day, cells were washed in PBS and cell media was replenished with 1.35 mL fresh media, 150 μL 10× lentiviral stock supernatant (MOI of approximately 6), and 4 μg/mL Polybrene (Sigma-Aldrich). Plates were incubated at 37°C with 5% CO_2_ for 24 hours. Subsequently, mKAte2-transduced cells were washed in PBS and expanded in culture until optimal confluency was achieved.

To isolate individual clones of transduced cells, fluorescent MDA-MB-231, NCI-H460 and LNCaP cells were singly sorted into individual wells of a 96 well plate (Corning) at the University of Michigan Flow Cytometry Core using a FACS Synergy Head #1 cell sorter (Sony Biotechnology). The viability marker Zombie Violet was used to exclude dead cells. The cells sorted were negative for Zombie Violet (450/50 (405)) and in the top 5% of mKate fluorescence intensity (615/30(561)).

To monitor tumor growth by bioluminescence imaging in vivo, MDA-MB-231 breast cancer cells were transduced with a luciferase lentivirus reporter regulated by the CMV promoter (purchased from the University of Michigan Vector Core). Transduction was carried out by centrifuging 2 × 10^6^ cells in 1 mL media (1,000*g* for 2 hours) with addition of 8 μg/mg Polybrene and 1 mL 10× luciferase virus (MOI of approximately 6). Culture media was replaced with fresh, warm media after 18 hours, and luciferase expression was analyzed 5 days later using a Dual-Luciferase Reporter Assay System (Promega) and bioluminescence imaging.

### Isolation of PBMCs.

Venous blood from healthy volunteers was collected in sterile anticoagulant vacuum tubes (BD Vacutainer sodium heparin). PBMCs were isolated using dextran sedimentation and Ficoll-Paque density-gradient separation (GE Healthcare). The gradient was then centrifuged at 400*g* for 15 minutes, and the buffy coat was collected and washed in PBS. Isolated PBMCs were resuspended at 1 × 10^7^ cells/mL in RPMI-1640 culture medium supplemented with 10% FBS. Viability was measured by trypan blue dye exclusion assay (Thermo Fisher Scientific). PBMCs were used directly for immune cell killing assays or were enriched for specific subpopulations. NK and CD8^+^ cells were purified from PBMCs using the EasySep Human NK Cell Isolation Kit and EasySep Human CD8 Positive Selection Kit, respectively (STEMCELL Technologies).

### Cancer cell killing assays.

Nuclear fluorescent MDA-MB-231 and LNCaP tumor cells were seeded in 96-well plates (Corning) at a density of 2 × 10^4^ cells per well and grown overnight. NCI-H460 tumor cells were plated at 2 × 10^3^ cells per well and also maintained overnight. On the day of the assay, PBMCs were isolated from healthy volunteers as previously described, and stocks of 1 × 10^6^ PBMCs/mL were separately incubated with 10 μg/mL of either UMCD6 or IgG isotype control antibodies directed against vWF or anti–LFA-1 for 1 hour at 37°C. Subsequently, 50 μL PBMC/antibody solution (50,000 PBMCs/well) and 50 μL caspase-3/7 reagent at 5 μM (Essen Bioscience) were added to each well.

Cells were imaged at 10-fold magnification in an IncuCyte S3 Live Cell Analysis System (Sartorius) at 37°C with 5% CO_2_. Images were acquired every 30 minutes or 1 hour for 5–7 days, 2–4 images per well. Data were analyzed using IncuCyte analysis software to detect and quantify the number of green (apoptotic) cells per image. A filter threshold of 100 μm^2^ was established to remove PBMC death events and other green fluorescent aberrations. The number of red events (survival of tumor cells) was calculated by counting red fluorescent mKate2-expressing nuclei. Data were plotted using GraphPad Prism software.

We also compared the effectiveness of UMCD6 versus the PD-1 inhibitors pembrolizumab and nivolumab. Similar to previous immune cell killing assays, MDA-MB-231 and NCI-H460 cell lines were seeded in 96-well plates at 2 × 10^4^ cells/well and 2 × 10^3^ cells/well, respectively. 1 × 10^6^/mL PBMCs, NK cells, and CD8^+^ cells were isolated and incubated in separate tubes with 10 μg/mL of UMCD6, pembrolizumab, nivolumab, or an IgG isotype control antibody for 1 hour at 37°C. 50 μL/well of the immune cell/antibody mixtures was layered over the MDA-MB-231 and NCI-H460 cells. 50 μL caspase-3/7 reagent (5 μM) were also added to each cancer well. Cells were imaged in the IncuCyte S3 Live Cell Analysis System as previously described.

### Subcutaneous xenograft study.

To assess the effects of UMCD6 in vivo, we injected SCID beige mice (Charles River) subcutaneously with 5 × 10^6^ luciferase-infected MDA-MB-231 cells. Mice were anesthetized through the i.p. route with a mixture of ketamine (80–120 mg/kg) and xylazine (5–10 mg/kg), and tumor cells were injected into the right flank of each mouse. Tumor growth was monitored by bioluminescence imaging. 26 days after cell implantation, when tumors had reached volumes of at least 100 mm^3^, 10 mice were randomly allocated to receive an intravenous injection of 1.2 × 10^7^ PBMCs via the tail vein, while the 3 other mice (control group) received PBS.

The following day, mice that had received PBMCs were divided into 2 groups, selected such that the range of tumor sizes was equal in the groups: 5 mice received a single dose of UMCD6 (400 μg/mouse) and 5 mice received an IgG control antibody (400 μg/mouse) i.p. Tumor growth was monitored every other day by bioluminescence imaging. To assess tumor volume via bioluminescence imaging, we i.p. injected mice with 100 μL sterile d-luciferin at 15 mg/mL (Gold Biotechnology) and anesthetized them with 2% isoflurane. Mice were then imaged with a Xenogen IVIS 200 bioluminescence camera following d-luciferin administration, and images were captured after 1 minute of exposure and then quantified using Living Image 2.60.1 software. All images were normalized to the same scale and exposure time.

### Internalization of UMCD6.

UMCD6 was directly labeled with cyanine 3 (Cy3), and successful antibody labeling was verified by flow cytometry. PBMCs from a healthy donor were stained with Cy3-conjugated UMCD6 at 4°C for 30 minutes. A Cy3-labeled CD45 antibody (BioLegend, clone 30F11) was used as control. Green fluorescence images were acquired through an IncuCyte S3 Live Cell Analysis System at 37°C every hour for 5 days, and internalization was measured by loss of green expression at the cell surface.

### Immunofluorescence histochemistry.

Xenograft tumors derived from MDA-MB-231 cells were dissected for histological examination. Tumors were harvested and placed in 4% PFA for at least 48 hours. Subsequently, tissues were embedded in ornithine carbamyl transferase (OCT) for cryosectioning at 8 μm using a Leica CM1950 cryostat.

For immunofluorescence staining, slides were fixed with 4% paraformaldehyde and blocked for nonspecific binding using 10% goat serum and 5% FBS in PBS (Millipore) for 2 hours at room temperature. Goat anti–human CD56 (BioLegend, clone 5.1H11) or goat anti–human CD3 (BioLegend, clone HIT3a) were incubated at 1:100 dilutions overnight at 4°C. After primary antibody incubation and washing, secondary goat anti-mouse IgG coupled to Cy3 antibody (Jackson ImmunoResearch Laboratories Inc.) was added to each slide for 1 hour at room temperature. To preserve fluorescence, samples were mounted using a DAPI solution containing ProLong Gold antifade and mounting medium (Invitrogen). Negative controls included isotype control antibodies. Fluorescence images were taken using an Olympus BX51 microscope equipped with a 40× lens objective (Olympus America Inc.). Images were captured with an Olympus DP-6 digital camera and processed with Adobe Photoshop 2020.

### Statistics.

Statistical analyses for the cancer killing assays were performed using GraphPad software. Data are shown as mean ± SEM, and statistical significance between 2 groups was determined by unpaired Student’s *t* test; a *P* value less than 0.05 was considered statistically significant. Total expression of NK receptors was calculated for each extracellular and intracellular marker by multiplying the mean fluorescence intensity of the positive cells by the percentage of positive cells. Data are presented as the ratio UMCD6-treated over IgG control at 0, 24, 48, and 72 hours. A paired 2-tailed *t* test was used to determine statistical significance; *n* = 6.

### Study approval.

Animal experiments were done in accordance with ethical guidelines and approved by the Animal Care and Use Committee at the University of Michigan (Immunopathogenesis of Malignancies and Chronic Inflammatory Disorders; PRO00008344). PBMCs from healthy volunteers were isolated in our laboratory following appropriate guidelines approved by the University of Michigan (Unique Surface Structures of Synovial Cells IRBMED 1988-0305; HUM00043197).

## Author contributions

JHR and DAF designed this study. MGR, JHR, and DAF contributed to drafting of the manuscript. MGR, MAA, and PLC performed in vivo experiments in mice. MGR, KSA, SMR, DPW, PR, RJG, and MEL performed and analyzed the cancer cell killing assays. MGR, PR, RJG, and MEL conducted immunohistochemical staining. MGR and QW designed, performed, and analyzed the NK and CD8 flow cytometry panels. TML provided the mKate2 2X nuclear localization fusion construct. VGK provided and analyzed the Affymetric data. PST, YMD, NGS, and FL helped with drafting of the manuscript and with the conceptual framework of this study.

## Supplementary Material

Supplemental data

Supplemental Video 1

Supplemental Video 2

Supplemental Video 3

## Figures and Tables

**Table 1:**
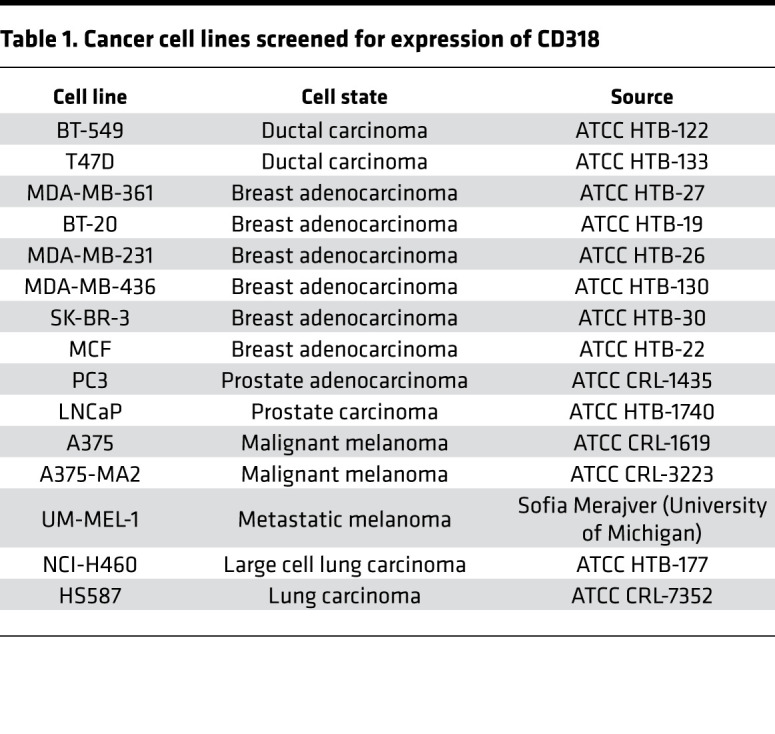
Cancer cell lines screened for expression of CD318

## Version 1 01/26/2021

In-Press Preview

## Version 2 03/08/2021

Electronic publication
