# Supplementary material for: CD6 is a target for cancer immunotherapy
Source: JCI Insight. 2021 Mar 8;6(5):e145662. doi: 10.1172/jci.insight.145662 (PMC8021120; doi:10.1172/jci.insight.145662)
Supplement: Supplemental data [file jciinsight-6-145662-s104.pdf]

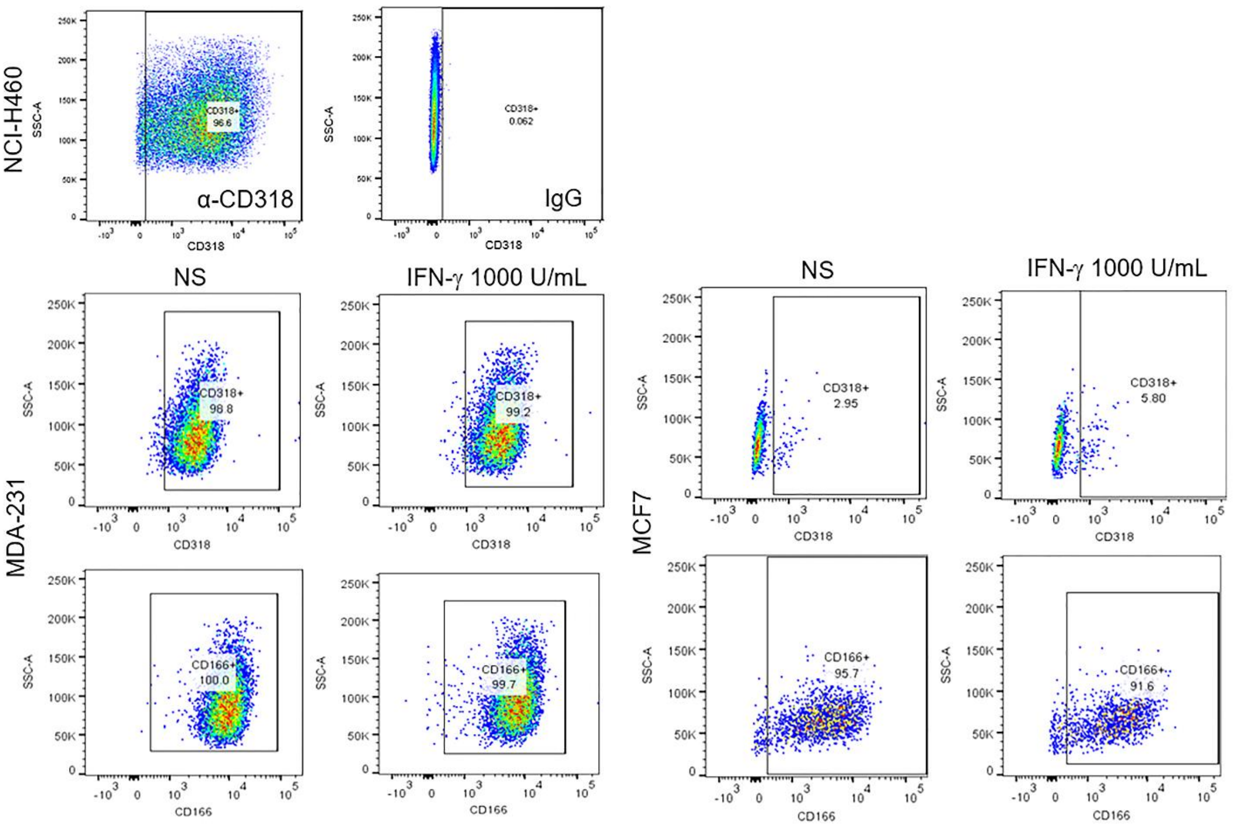

Supplementary figure1. NCI-H460 (NSCLC) cells express CD318. Upper panel: FACs analysis on NCI-H460 lung cancer cells revealed that CD318 is expressed on nearly all of the tumor cells (96.6%). Cell surface CD318 expression was evaluated using a mouse anti-human CD318 antibody (Miltenyi Biotec) at 1:100 dilution with $5x10^5$ cells in FACs buffer. Representative negative control histogram using NCI-H460 NSCLC cells shows 0.062% positivity. FcR Blocking Reagent was added to all cells to block non-specific antibody binding to cancer cells (Miltenyi Biotec). Similar background fluorescence was obtained using IgG control antibody for all cell lines evaluated for CD318 expression. Lower panels: MDA, but not MCF7 HBCCs, express CD318. FACs analysis on MDA (lower left panel) and MCF7 (lower right panel) HBCCs revealed that CD318 is expressed on nearly all of the MDA cells but on 10%< of MCF7 cells. The addition of IFN-γ (1000U/mL) did not significantly alter MDA or MCF7 CD318 expression in vitro. Notably, CD166 (ALCAM) an alternative CD6 ligand, was highly expressed on MDA-231 (NS: 100%; IFN-γ: 99.7%) and MCF7 (NS: 95.7%; IFN-γ: 91.6%) HBCCs regardless of stimulation with IFN-γ. NS is no stimulus.

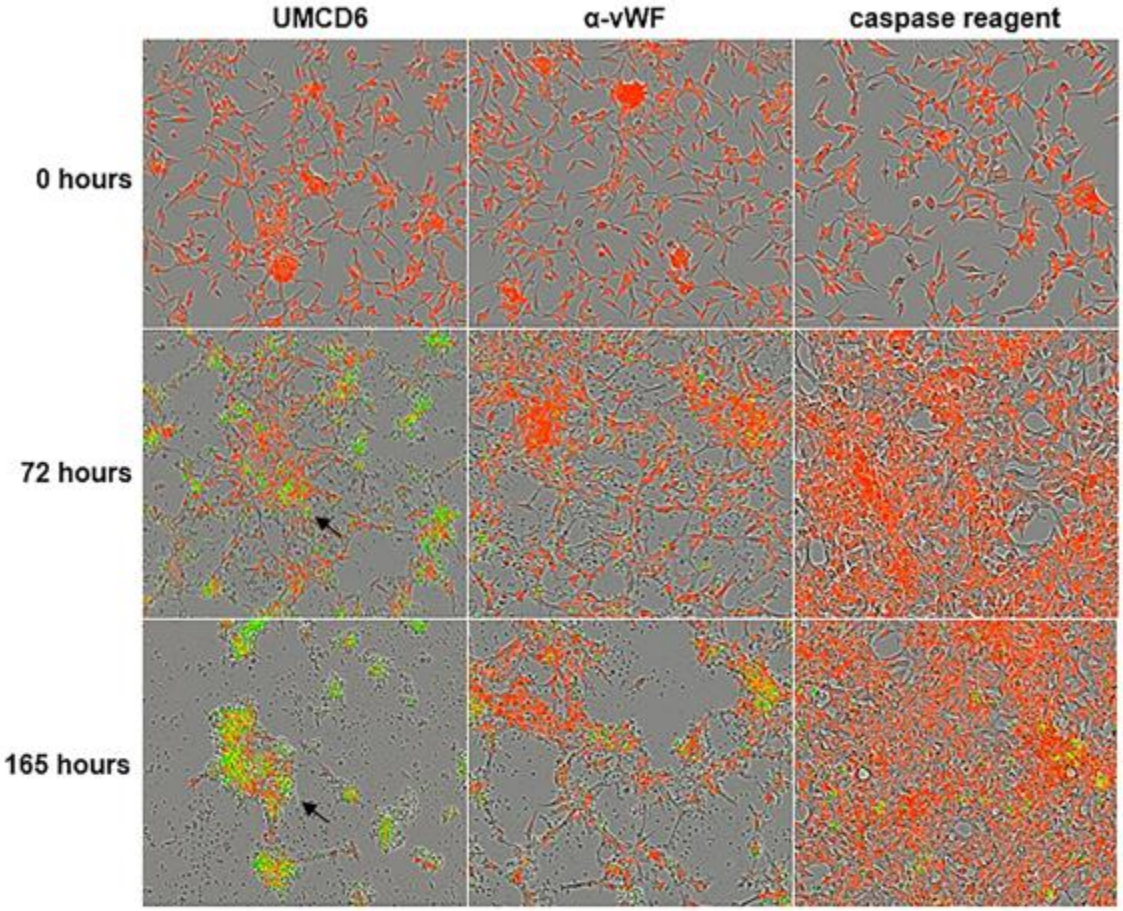

Supplemental Figure S2. UMCD6 enhances LNCaP prostate cancer cell killing in vitro by human PBMCs. In the absence of mAbs and/or PBMCs (right column – untreated wells), LNCAP proliferation was unimpeded (fluorescent red cells). LNCAP prostate cancer cells co-cultured with UMCD6-treated PBMCs displayed profound clumping and caspase expression (fluorescent green dye) at 72 hours and were almost completely eliminated by 165 hours (see arrows). In the presence of control antibody and PBMCs modest killing of LNCaP cells was observed, but viable cancer cells persisted. The experiment was performed using the IncuCyte imaging device. Additional information: the video can be found in the multimedia section of the online article.

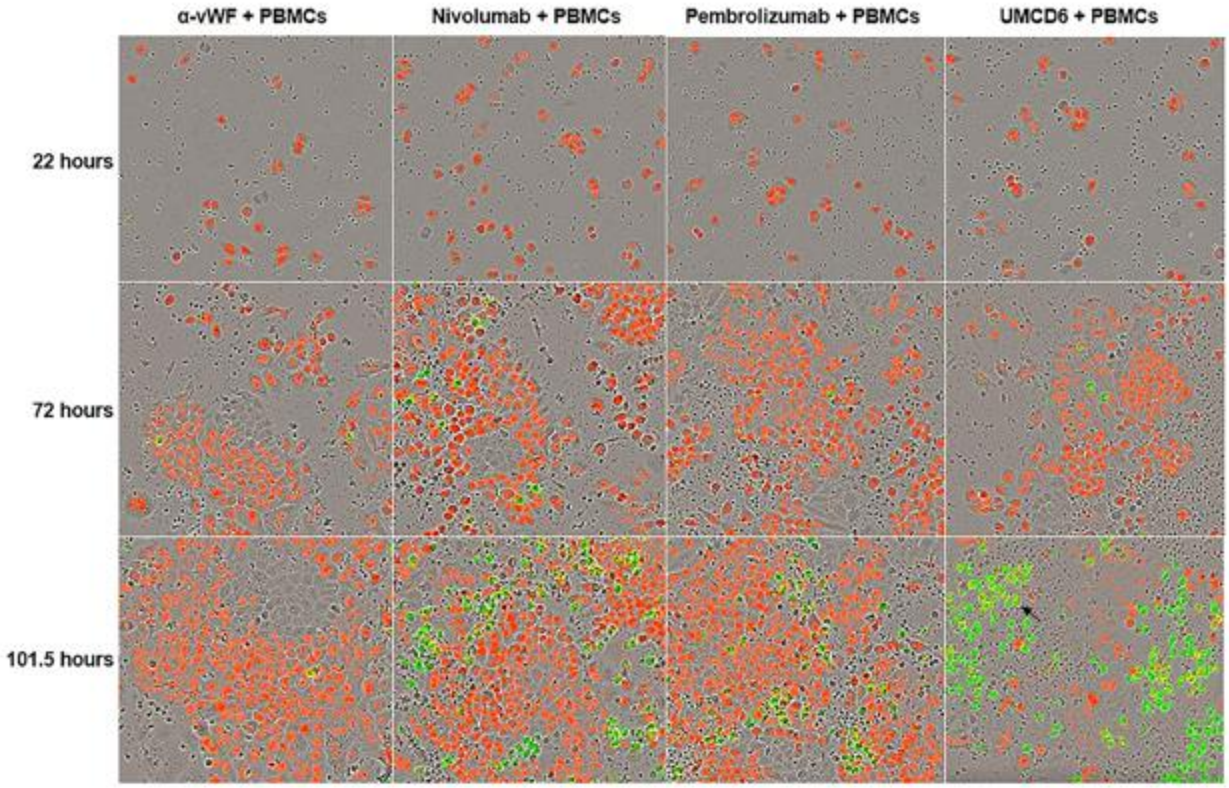

Supplemental Figure S3. Time course of UMCD6 mediated killing of NSCLC cells (NCI-H460) by human PBMCs. CD318 expressing NCI-H460 cells were plated in a 96-well plate with a seeding density of 2,000 cells per well. Non-activated PBMCs (35,000) were added to the tumor cells at about 22 hours. Before addition to the co-cultures PBMCs were incubated for an hour with either UMCD6 (mouse anti-human CD6) or mouse anti-human vWF (a non-specific IgG control antibody) or nivolumab or pembrolizumab (10µg/mL). Tumor cell killing was measured in an IncuCyte cell imaging device by evaluating the number of NCI-H460 cells present in each well expressing nuclear caspase (green fluorescence). NCI-H460 cells in co-cultures with UMCD6 showed profound clumping and caspase expression after 101.5 hours (see arrow) compared co-cultures with other treatments. Notable loss of red fluorescing NSCLC tumor cells – indicating inhibited growth and cell survival can be seen starting at about 3 days in co-cultures treated with UMCD6. Additional information: the video can be found in the multimedia section of the online article.

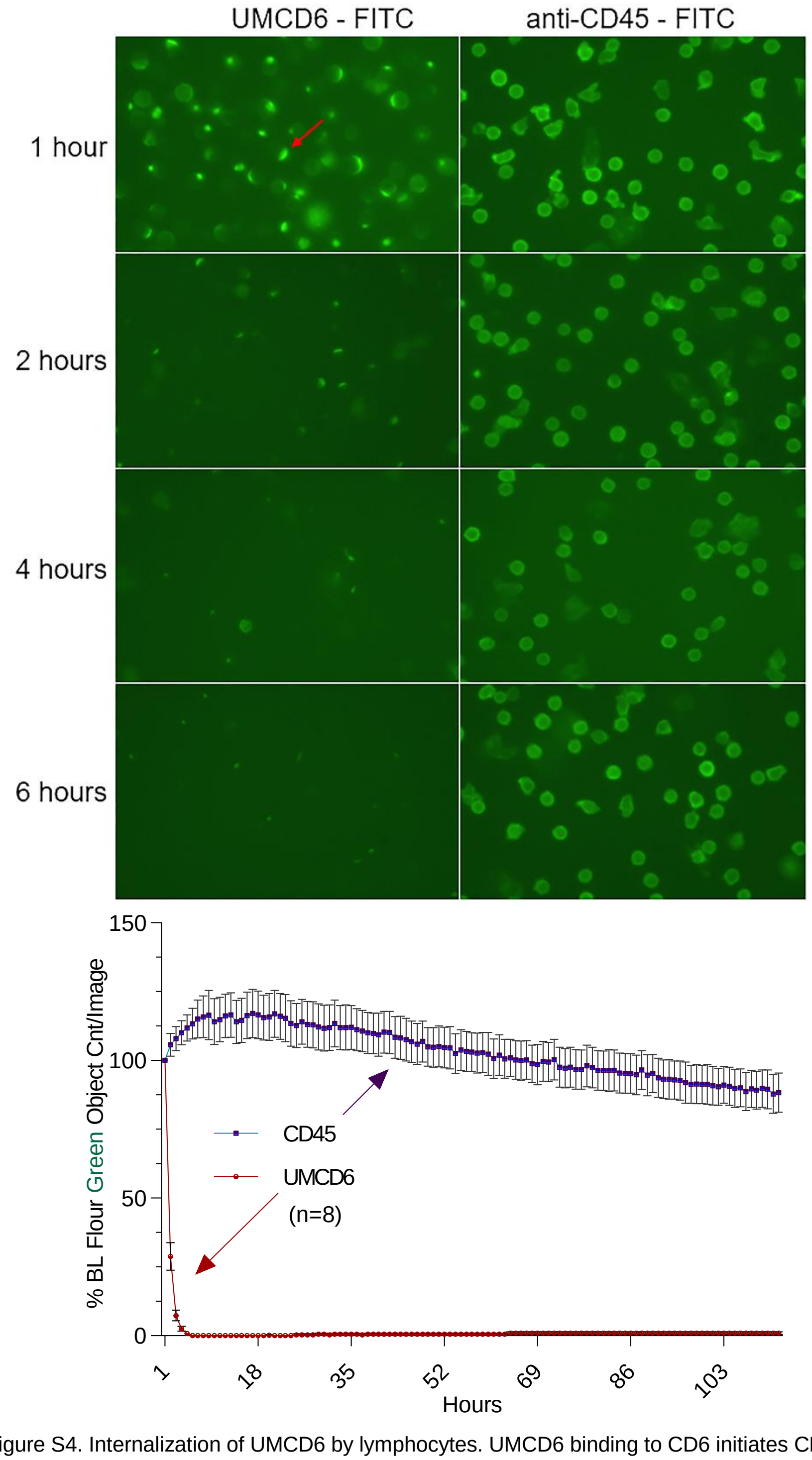

Supplemental Figure S4. Internalization of UMCD6 by lymphocytes. UMCD6 binding to CD6 initiates CD6 capping and complete internalization from the cell surface of within 6 hours (see arrow). Human peripheral blood lymphocytes were incubated with either Cy3-labeled UMCD6 or anti-CD45 at 4°C for 30 minutes and fluorescent images were taken at 40X magnification at 1, 2, 4 and 6 hours. Upper panel: fluorescent green images show capping of CD6 on most cells at 1 hour, followed by internalization of UMCD6 with loss of green fluorescence on the cell surface. Lower panel: CD6 and CD45 surface expression on PBMCs were analyzed by the green object count per image through the IncuCyte imaging system, compared to the count at time 1 hour, which was set at 100%. The graph shows that the percentage of CD6+ fluorescent cells decreased rapidly to 0% over 6 hours, while CD45 surface staining remained nearly constant (90-95%) for 5 days (n=8).

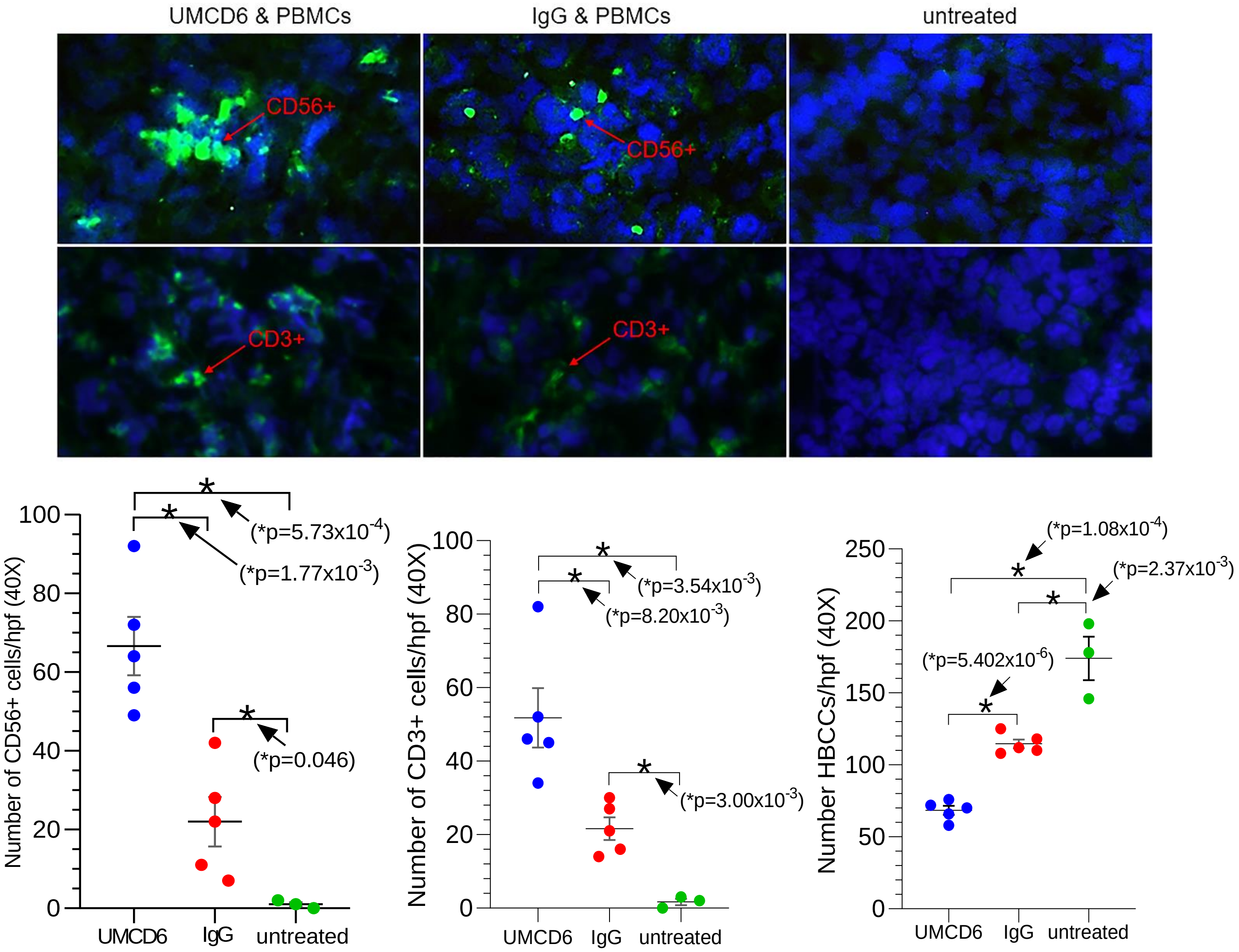

Supplemental Figure S5. Accumulation of lymphocytes in MDA-MB-231 tumors xenotransplanted into SCID beige mice following infusion of human PBMCs and UMCD6. Tumors were removed at day 36 after injection of tumor cells into the flanks of SCID beige mice, day 10 after infusion of $12x10^6$ PBMCs, and day 9 after intraperitoneal injection of 0.4 mg UMCD6 or control IgG. Tumors were cryosectioned and immunostained for CD56 and CD3. Upper panel: Tumor tissues immunostained for CD56 (human NK cell marker) or CD3 (human lymphocyte marker) from mice administered UMCD6 showed activated NK cells that were associated with areas of decreased density of tumor cells (40X). CD3+ lymphocyte staining revealed results similar to the NK cell staining. DAPI stain is shown in fluorescent blue. Lower panel: Numbers of CD56+ NK cells, CD3+ T lymphocytes and tumor cells were evaluated by counting the numbers of green or red fluorescing cells/hpf. MDA cells were fluorescent red due to expression of a transfected RFP. The numbers of CD56+ and CD3+ cells/hpf were significantly higher in the UMCD6-treated mice (CD56: 1 section/tumor from n=5 mice for UMCD6 and IgG, and 1 section/tumor from n=3 mice for untreated), (CD3: 1 section/tumor from n=5 mice for UMCD6 and IgG, and 1 section/tumor from n=3 mice for untreated) whereas the number of tumor cells was significantly decreased compared to mice that received either control IgG + PBMCs or no treatment (1 section/tumor from n=5 mice for UMCD6 and IgG, and 1 section/tumor from n=3 mice for untreated).

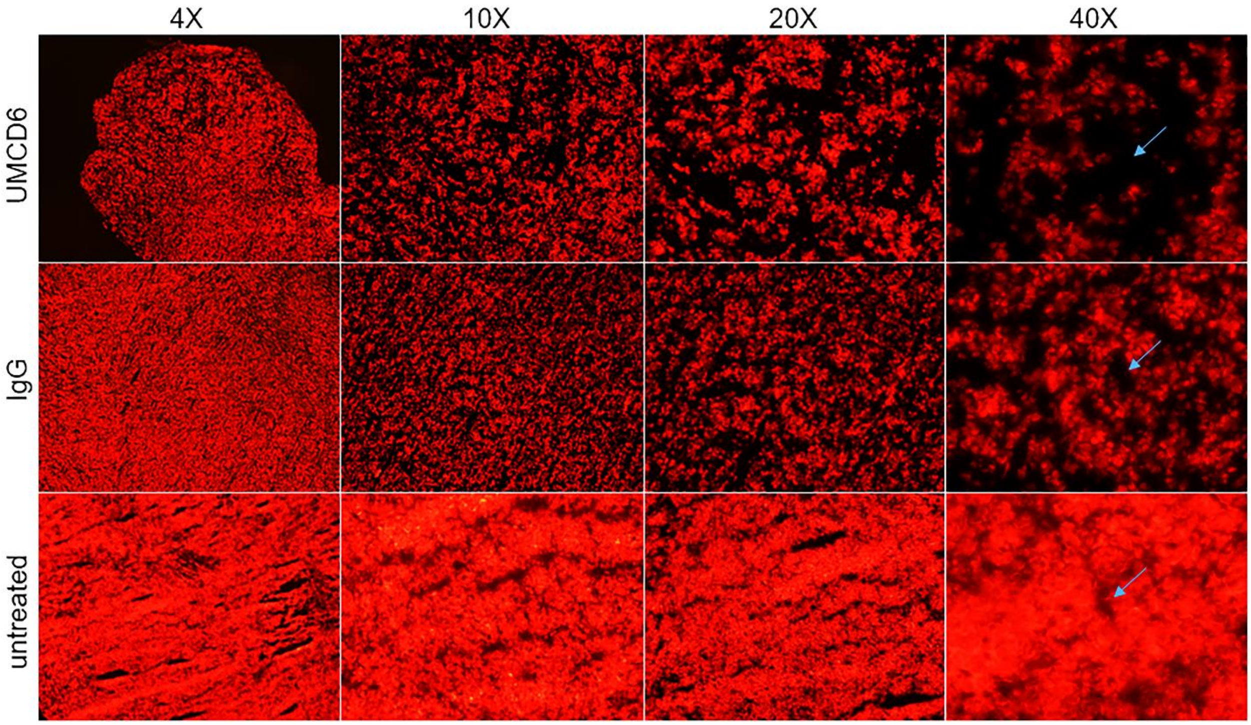

Supplemental Figure S6. MDA-MB-231 xenografts are reduced with UMCD6 treatment. Tumor cell density in MDA-MB-231 xenografts are shown. Upper panel: representative images of endogenous red fluorescent tumor cells in each xenografted tumor harvested 10 days after PBMC infusion. Fewer mKate2-transduced MDA-MB-231 cells are seen at 40x magnification in mice receiving UMCD6 compared to mice receiving IgG control antibody or in untreated mice. Notable differences in tumor sizes among the different groups can be seen at 4X. UMCD6 treated mice exhibited tumors with large gaps in the tumor microenvironment that were not observed in the IgG control and untreated mice (see arrows).

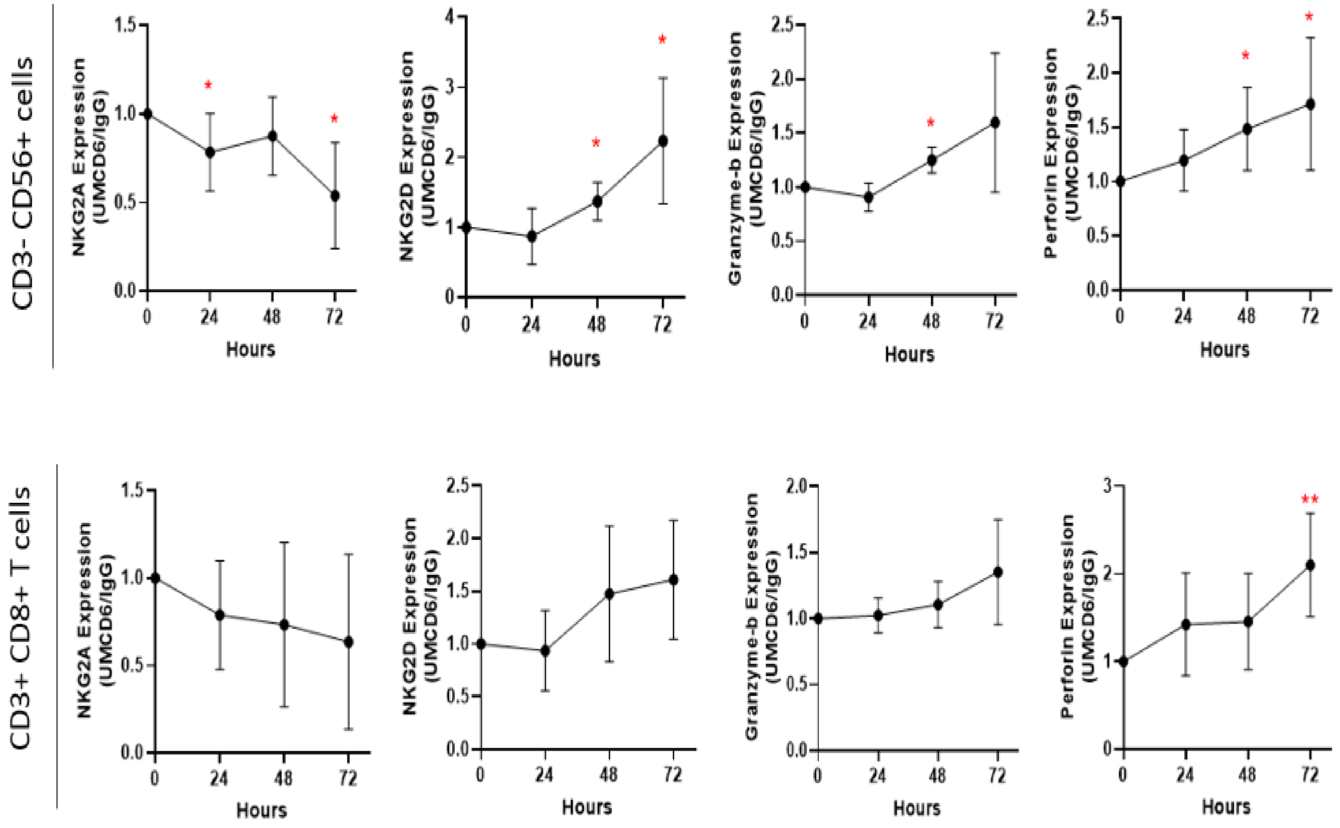

Figure S7. Effect of UMCD6 on the expression of NKG2A, NKG2D, Granzyme-b and perforin by NK and CD8+ T cells. Expression levels of the inhibitory receptor NKG2A, activating receptor NKG2D, cytotoxic serine protease Granzyme-b, and pore-forming protein perforin were studied by flow cytometry. Total expression was calculated for each extracellular and intracellular marker by multiplying the mean fluorescence intensity of the positive cells by the percentage of positive cells. Data are presented as the ratio UMCD6-treated over IgG control at 0, 24, 48 and 72 hours. A paired t-test was used to determine statistical significance, *$p<0.05$, n=6. Upper panels: upon activation with UMCD6, NK cells upregulated the expression of NKG2D by 48 and 72 hours, whereas the expression of NKG2A was downregulated by 24 and 72 hours ($p<0.05$). Granzyme-b was upregulated by 48 hours (*$p<0.05$) and perforin expression was upregulated at 48 and 72 hours ($p<0.05$). Lower panels: UMCD6 effect on CD8+ T cells reveals a statistically significant increase in the expression of perforin ($p<0.05$) at 72 hours (n=6).
